# Comparative Evolutionary and Developmental Dynamics of the Cotton (*Gossypium hirsutum*) Fiber Transcriptome

**DOI:** 10.1371/journal.pgen.1004073

**Published:** 2014-01-02

**Authors:** Mi-Jeong Yoo, Jonathan F. Wendel

**Affiliations:** 1 Department of Biology, University of Florida, Gainesville, Florida, United States of America; 2 Department of Ecology, Evolution, and Organismal Biology, Iowa State University, Ames, Iowa, United States of America; Harvard University, United States of America

## Abstract

The single-celled cotton (*Gossypium hirsutum*) fiber provides an excellent model to investigate how human selection affects phenotypic evolution. To gain insight into the evolutionary genomics of cotton domestication, we conducted comparative transcriptome profiling of developing cotton fibers using RNA-Seq. Analysis of single-celled fiber transcriptomes from four wild and five domesticated accessions from two developmental time points revealed that at least one-third and likely one-half of the genes in the genome are expressed at any one stage during cotton fiber development. Among these, ∼5,000 genes are differentially expressed during primary and secondary cell wall synthesis between wild and domesticated cottons, with a biased distribution among chromosomes. Transcriptome data implicate a number of biological processes affected by human selection, and suggest that the domestication process has prolonged the duration of fiber elongation in modern cultivated forms. Functional analysis suggested that wild cottons allocate greater resources to stress response pathways, while domestication led to reprogrammed resource allocation toward increased fiber growth, possibly through modulating stress-response networks. This first global transcriptomic analysis using multiple accessions of wild and domesticated cottons is an important step toward a more comprehensive systems perspective on cotton fiber evolution. The understanding that human selection over the past 5,000+ years has dramatically re-wired the cotton fiber transcriptome sets the stage for a deeper understanding of the genetic architecture underlying cotton fiber synthesis and phenotypic evolution.

## Author Summary

Ever since Darwin biologists have recognized that comparative study of crop plants and their wild relatives offers a powerful framework for generating insights into the mechanisms that underlie evolutionary change. Here, we study the domestication process in cotton, *Gossypium hirsutum*, an allopolyploid species (containing two different genomes) which initially was domesticated approximately 5000 years ago, and which primarily is grown for its single-celled seed fibers. Strong directional selection over the millennia was accompanied by transformation of the short, coarse, and brown fibers of wild plants into the long, strong, and fine white fibers of the modern cotton crop plant. To explore the evolutionary genetics of cotton domestication, we conducted transcriptome profiling of developing cotton fibers from multiple accessions of wild and domesticated cottons. Comparative analysis revealed that the domestication process dramatically rewired the transcriptome, affecting more than 5,000 genes, and with a more evenly balanced usage of the duplicated copies arising from genome doubling. We identify many different biological processes that were involved in this transformation, including those leading to a prolongation of fiber elongation and a reallocation of resources toward increased fiber growth in modern forms. The data provide a rich resource for future functional analyses targeting crop improvement and evolutionary objectives.

## Introduction

Ever since Darwin's time, biologists have recognized that human domestication of wild plants and animals offers promising opportunities for enhancing our understanding of the evolutionary process. As highlighted in recent reviews [1], [2], comparisons among wild and domesticated forms of crop plants often lead to insights into the genetic architecture and developmental mechanisms that underlie traits subjected to strong directional human selection. The power of this approach is magnified by the recent advent of high-throughput “omics” technologies, which hold promise for leading us to an eventual systems-level understanding of phenotypic change. Domesticated forms of cultivated species differ from their wild counterparts in numerous traits, particularly those subjected to intentional directional selection, e.g., loss of seed dormancy, larger and/or more fruits, determinate growth, annualized habit, and earlier flowering. Insights into the evolution of this “domestication syndrome” [3] are made possible by comparative studies of wild and domesticated representatives of individual cultivated species [1], [2].

Upland cotton (*Gossypium hirsutum* L.) is the most important domesticated fiber plant in the world, accounting for more than 90% of global cotton production. Originally native to the northern coast of the Yucatan peninsula in Mexico, upland cotton is widely cultivated in over 50 countries in both hemispheres [4]. The trait for which cotton was initially domesticated is the remarkably elongated, single-celled epidermal trichomes, or hairs, that cover the cottonseed surface (colloquially termed “fibers”). These seed hairs vary greatly in length, color, strength, and density among the myriad wild, semi-domesticated, feral and modern annualized forms that collectively comprise the species *G. hirsutum*. In truly wild *G. hirsutum* trichomes are short (typically <1.5 cm), coarse, and are various shades of tan to brown. *Gossypium hirsutum* was initially domesticated at least 5000 years ago, and following millennia of directional selection, domesticated forms now produce long, strong, and fine white fibers along with a dramatically enhanced fiber yield. In addition to this increase in fiber length, strength, and quality, the domestication process brought about other morphological transformations, including decreased plant stature, earlier flowering, and loss of seed dormancy.

*Gossypium hirsutum* is an allotetraploid containing two diverged sets of chromosomes, “A” and “D”, which became reunited in a common nucleus as a result of a hybridization event approximately 1–2 million years ago (mya). This merger of an African/Asian, A genome (similar to modern *G. arboreum*) and an American, D genome (much like modern *G. raimondii*) gave rise to a new allopolyploid lineage that diversified into five species (AD_1_ to AD_5_) [4], [5]. Considering the importance of polyploidy as a major evolutionary process in plants and its prevalence in all flowering plants [6], comparative analyses of wild and domesticated cottons may provide new perspectives about how human selection affects duplicated genes in allopolyploids. In addition, many important crops, such as alfalfa, potato, wheat, soybean, and cabbage, are obvious polyploids, so studying gene expression in allopolyploid cotton has the potential to offer novel insights on the role of polyploidy in crop evolution (e.g., Bao et al.[7]).

To date, and despite its importance to understanding molecular mechanisms governing fiber development, there have been only a handful studies of global gene expression in *G. hirsutum*, using expressed sequence tags (ESTs) [8], [9] and microarrays [10]–[14]. In addition, most have focused on comparisons of modern, annualized *G. hirsutum* and its fiberless/lintless mutants. The single notable exception is the study of Rapp et al., who used microarrays to compare truly wild and domesticated *G. hirsutum* [13]. Notably, Rapp et al. explored global gene expression patterns in wild and domesticated *G. hirsutum* cotton fibers across five temporal/developmental time points, and found that about one quarter of all genes examined exhibited expression changes during domestication, indicating massive alteration of the cotton fiber transcriptome by domestication and crop improvement [13]. However, a limitation of the study of Rapp et al. is that they employed only one accession representing each of the wild and domesticated gene pools, raising the possibility that some of the observed differential expression might simply reflect expression variation that is unconnected to the evolutionary transformation of interest [13]. Also, the microarray methodology relies on less precise probe/target hybridization, is subject to high background noise, and has a narrower range of gene expression quantification, in comparison to profiling using RNA-Seq data [15]. Finally, the genome sequence for *G. raimondii* only recently became available [16], providing deeper annotation and better discrimination among homologs (and homoeologs), and hence enhanced power to decipher gene expression level changes across the whole genome.

Here, to gain insight into the evolutionary genomics of cotton domestication, we conducted comparative transcriptome profiling of developing cotton fibers from multiple accessions of wild and domesticated *G. hirsutum* using RNA-Seq data. Two developmental stages were studied, 10 and 20 days post anthesis (dpa), representing key stages of primary cell wall growth and the transition to secondary cell wall growth, respectively [17], [18]. By examining gene expression levels digitally, we found that approximately one-third of the genes in the genome are expressed in cotton fiber regardless of lineage, accession, and developmental stages. Notably, nearly 5,000 genes are diagnosed as being differentially expressed as a consequence of cotton fiber domestication. These data suggest that human selection has reprogrammed the transcriptome on a massive scale, and that part of this rewiring entails a reallocation from stress response pathways toward fiber growth.

## Results

We performed global transcriptome profiling of developing cotton fibers from wild and domesticated *G. hirsutum* using RNA-Seq. A total of 310 million (M) reads was generated from 20 libraries, and on average 70% of these uniquely mapped to the reference genome (Table 1). To determine how many genes were expressed in fibers and whether there was variation among accessions, we first evaluated the number of expressed genes. Since we did not include external controls, such as the External RNA Controls Consortium (ERCC) controls, we used arbitrary measures for “expression”, such as RPKM = 2 or 5 ( = 32 or 80 short reads on average for a 1.6 kb-gene; RPKM = Reads Per Kilobase of gene model per Million mapped reads) [19]. Based on the criterion of RPKM≧5, approximately 12,700 (33.9%) and 12,000 (33.0%) genes were expressed at 10 and 20 dpa, respectively, in most accessions (Table 2); three domesticated accessions (Cascot L-7, Coker 315, and CRB250) showed lower numbers of expressed genes at 20 dpa compared to other accessions, which we attribute to the higher proportions of redundantly mapped reads in these accessions (data not shown). This is consistent with the fiber transcriptome diversity obtained from domesticated *G. hirsutum* cv. TM-1 [20] and from diploid cotton *G. arboreum* [21]. In general, more genes were expressed in wild than domesticated cottons at both developmental stages. More genes were expressed at 20 dpa than 10 dpa in wild cottons, while the opposite was observed for domesticated cottons (Table 2). Before identifying differentially expressed genes during domestication and development, variation among samples was evaluated using Multidimensional scaling (MDS). Because the transcriptome profile from Maxxa 10 dpa exhibited a large distance from other domesticated cottons and was embedded in wild cottons (Figure S1), it was excluded from further analyses. However, in general, samples clustered as expected, indicating that the variation among samples is largely explained by developmental stage and domestication.

**Table 1:** Accession information used in this study and the number and percentage of short reads mapped onto the Cotton D genome reference assembly.

Species	Origin	Stage	Total Reads	Mapped Reads (%)
*G. hirsutum* var. *yucatanense* (TX2090)	Yucatan, Mexico	10 dpa 20 dpa	9,598,358 10,517,180	7,554,107 (78.7) 8,175,498 (77.7)
*G. hirsutum* var. *yucatanense* (TX2094)	Yucatan, Mexico	10 dpa 20 dpa	11,139,818 14,110,893	8,326,479 (74.7) 10,627,792 (75.3)
*G. hirsutum* var. *yucatanense* (TX2094 = YUC*)	Yucatan, Mexico	10 dpa 20 dpa	10,444,338 10,991,115	8,908,976 (85.3) 7,851,707 (71.4)
*G. hirsutum* var. *yucatanense* (TX2095)	Yucatan, Mexico	10 dpa 20 dpa	42,989,086 15,142,099	33,550,251 (78.0) 11,646,705 (76.9)
*G. hirsutum* var. *palmeri* (TX665)	Yucatan, Mexico	10 dpa 20 dpa	14,559,567 29,461,705	11,627,366 (79.9) 23,229,997 (78.8)
*G. hirsutum* cv. Cascot L-7	Plains in U.S.A	10 dpa 20 dpa	10,688,995 31,345,469	8,488,820 (79.4) 17,785,760 (56.7)
*G. hirsutum* cv. Coker 315	Eastern U.S.A	10 dpa 20 dpa	14,201,314 10,615,133	11,065,757 (77.9) 5,881,452 (55.4)
*G. hirsutum* cv. CRB252†	Eastern U.S.A	10 dpa 20 dpa	9,986,108 8,344,787	7,583,439 (75.9) 4,720,388 (56.6)
*G. hirsutum* cv. Maxxa*	Western U.S.A	10 dpa 20 dpa	12,266,868 15,484,446	9,410,233 (76.7) 12,353,194 (79.8)
*G. hirsutum* cv. Texas Marker 1 (TM1)	Delta in U.S.A.	10 dpa 20 dpa	14,706,919 13,078,240	11,766,434 (80.0) 9,003,797 (68.8)

CRB252-Derives from a double cross, SG 248/PHY 72//ST 474/Maxxa.

data from the study number SRP001603 at NCBI SRA. Fiber samples from TX2094 and Maxxa were collected in different growing seasons; thus, this TX2094 was noted as “YUC” to be distinguished from TX2094, which was collected in the same year as the other materials.

**Table 2:** The number of genes expressed in *Gossypium hirsutum* and *G. arboretum*.

Accession	Tissue*	domestication	RPKM≧2 (%)	RPKM≧5 (%)	N1†	Reference
TX665	10	wild	17,724 (47.3)	12,501 (33.3)		This study
TX2090	10	wild	18,846 (50.2)	13,674 (36.5)		
TX2095	10	wild	17,741 (47.3)	12,383 (33.0)		
TX2094	10	wild	19,057 (50.8)	13,849 (36.9)		
YUC	10	wild	20,396 (54.4)	15,096 (40.3)		
Cascot L-7	10	domesticated	15,977 (42.6)	11,150 (29.7)		
Coker 315	10	domesticated	16,885 (45.5)	11,852 (31.6)		
CRB 252	10	domesticated	17,264 (46.0)	12,260 (32.7)		
TM1	10	domesticated	16,544 (44.1)	11,884 (31.7)		
Maxxa	10	domesticated	17,465 (46.6)	12,055 (32.1)		
TX665	20	wild	18,487 (49.3)	13,068 (34.8)		This study
TX2090	20	wild	19,850 (52.9)	15,033 (40.1)		
TX2095	20	wild	19,393 (51.7)	14,094 (37.6)		
TX2094	20	wild	20,396 (54.4)	15,026 (40.1)		
YUC	20	wild	19,877 (53.0)	14,660 (39.1)		
Cascot L-7	20	domesticated	13,154 (35.1)	7,900 (21.1)		
Coker 315	20	domesticated	10,804 (28.8)	6,640 (17.7)		
CRB 252	20	domesticated	14,223 (37.9)	8,674 (23.1)		
TM-1	20	domesticated	17,212 (45.9)	11,730 (31.3)		
Maxxa	20	domesticated	16,652 (44.4)	11,506 (30.7)		
TM-1†	2	domesticated			11,094 (29.6)	[20]
TM-1†	7	domesticated			10,278 (27.4)	
TM-1†	10	domesticated			10,814 (28.8)	
TM-1†	20	domesticated			11,078 (29.5)	
TM-1†	25	domesticated			10,716 (28.6)	
TM-1†	various§	domesticated			11,913 (31.8)	
TX2094	young leaf	wild	18,794 (50.1)	11,350 (32.5)		[55]
Maxxa	young leaf	domesticated	19,618 (52.3)	12,183 (32.5)		
*G. arboreum*	10 to 24	domesticated diploid		12,227 (32.6)	[21]	

numbers indicate day post anthesis (dpa) fibers.

denotes the number of genes expressed at levels significantly different from zero (P<0.01) [20].

leaves, stems, petals, anthers, calyx, and bracts.

### Transcriptomic change during development in wild and domesticated *G. hirsutum*

**Figure 1:**
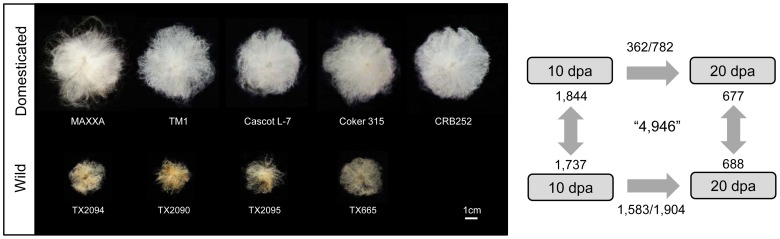
Number of genes differentially expressed during fiber development within and between wild and domesticated cottons. Left: Representative images of individual seeds with attached fiber are presented for domesticated (top) and wild (bottom) accessions. Right: Number of differentially expressed genes in developing cotton fiber within and between wild and domesticated cottons (RPKM≧1, FDR<0.05, fold-change≧1.5). For example, between two developmental stages within domesticated cottons, 362 genes were up-regulated at 10 dpa, whereas 762 genes were more highly expressed at 20 dpa. Similarly, between wild and domesticated cottons at 10 dpa, 1,844 genes were up-regulated in domesticated cottons, while 1,737 genes were more highly expressed in wild cottons.

We profiled the transcriptome during development in wild and domesticated cottons using two developmental stages, 10 and 20 dpa. Both wild and domesticated cottons showed more gene up-regulation than down-regulation during the transition from 10 to 20 dpa, e.g., 782 vs. 362 in domesticated cottons (Figure 1). However, three times as many genes (3,487 vs. 1,144) were differentially expressed during development in wild cottons compared to domesticated cottons (Figure 1). This pattern is also supported by the MDS plot, which showed less variation between the two developmental stages of domesticated cottons compared to wild cottons (Figure S1). Our results with respect to developmental variation differ from those of Rapp et al. [13], where differential expression was observed at 2.6-times as many genes in domesticated cotton relative to a wild accession (5,851 vs. 2,207 with 1.5-fold change; Table S1). However, our results parallel those from a second domesticated allopolyploid, *G. barbadense*, using either microarrays [22] or RNA-Seq data (M.J. Yoo et al., unpublished data). To clarity the difference between the two studies, we reanalyzed our data using the same accessions used in Rapp et al. [13]. The inclusion of TX2094 and YUC as two biological replicates resulted in 10 times as many DE genes compared to a single accession analysis (TX2094 vs. “TX2094+YUC” = 436 vs. 4,520; Table S1) and a 30% increase in DE genes relative to the “All accession” analysis (4,520 vs. 3,487; Table S1). As for domesticated cottons, since we have only one replicate for TM1, we included a second domesticated cotton for comparison, which resulted in a 17∼21% increase compared to the “All accession” analysis (Table S1).These results suggest that the observed conflict between the two studies likely is explained by technical differences among platforms and the reference genome used.

As expected based on our understanding of primary and secondary cell wall biosynthesis in cotton [17], [18], the two developmental stages were clearly differentiated by the expression patterns of genes involved in cell wall biogenesis. Cellulose synthase (*CesA*) genes, such as *CesA4*, *CesA6*, *CesA7*, and *CesA8* were more up-regulated at 20 than 10 dpa in wild cottons, while they exhibited less differential expression during fiber development in domesticated cottons (Figure S2A, B). Among them, four *CesA* genes were highly expressed at 20 dpa in domesticated *G. hirsutum*, consistent with a previous report [23], but only *CesA8*, a homologue of *GhCesA1*, was up-regulated at 20 dpa compared to 10 dpa in domesticated cottons (Figure S2A, B). Cellulose synthase-like (*Csl*) genes, particularly *CslA* and *CslC*, responsible for glucomannan and xyloglucan synthesis, respectively [24], were up-regulated at 10 dpa in both wild and domesticated accessions (Figure S2A, C). Additional differentially expressed genes related to cell wall biogenesis exhibited various patterns during fiber development. For example, β-galactosidase was up-regulated at 10 dpa, while β-1,3-glucosidase and β-xylosidase were up-regulated at 20 dpa; these two enzymes are thought to function in hydrolyzing galactan, glucan, and xylogucan, respectively, into monosaccharides, such as glucose, which can be further processed to either cellulose or pectin [25]–[27]. Xyloglucan endotransglycosylase (*XTH*) genes, which encode proteins involved in xyloglucan breakdown and subsequent rejoining with different acceptor chains, showed variable expression patterns during development (Figure S3). For example, *XTH5* and *XTH28* were up-regulated at 10 and 20 dpa, respectively, in both wild and domesticated cottons. Genes related to pectin synthesis, for example, UDP-D-glucuronate-4-epimerase, β-galactosidase, and pectate lyase, were also up-regulated at 10 dpa relative to 20 dpa in both wild and domesticated accessions. However, UDP-glucose-6-dehydrogenases, which oxidize UDP-glucose into UDP-glucuronate, were up-regulated at 10 dpa of domesticated cottons, but down-regulated in wild cottons. Since the foregoing genes represent only a small portion of the total number of differentially expressed genes, we further investigated the difference in development between wild and domesticated cottons using functional analyses (see below).

### Transcriptomic changes accompanying domestication

To investigate transcriptomic changes in cotton fiber that distinguish wild from domesticated cottons, and hence reflect the presumptive effects of human selection, we compared the gene expression patterns of wild and domesticated cottons from multiple accessions. A total of 4,946 (13.2%) genes were differentially expressed between wild and domesticated cottons (Figure 1), approximately evenly split between genes that were differentially up- and down-regulated between these two pools. However, nearly three times as many genes were differentially expressed at 10 relative to 20 dpa, a result that at least partially mirrors the data in Rapp et al. [13] (1.7-fold more genes differentially expressed at 10 dpa relative to 20 dpa); the two studies differ in that there was a greater bias toward up-regulation in domesticated than in wild cotton in the earlier study. However, if strict criteria for differential expression are applied, such as RPKM≧5 and more than 2-fold change (All accessions (RPKM> = 5) and Rapp et al. in Table S2), the two studies yield similar results; for example, about 60% of differentially expressed genes at both developmental time points were up-regulated in domesticated cottons relative to wild cottons, although it looks like there are more differentially expressed genes at 20 dpa in domesticated cottons than in wild cottons in Rapp et al. [13] compared to this study (not statistically significant; P = 0.1106) (Table S1). A single accession analysis resulted in an extremely small number of DE genes (<1% of the genes in the reference genome), perhaps due to the lack of biological replicates, while inclusion of two TX2094 samples in RNA-Seq data showed more DE genes compared to multiple accession analysis ((TX2094+YUC)−TM1 vs. All accession = 3,609 vs. 2,910 at 10 dpa, 3,299 vs. 1,339 at 20 dpa; Table S2). These results suggest that variation among biological replicates plays an important role in analyzing RNA-Seq data; that is, more biological replicates from one accession increase the power of DE gene detection (see the previous section). However, at the same time, including multiple accessions facilitates discovery of DE genes across multiple accessions (e.g., (TX2094+TX665+TX2095)−(TM1+CRB250+cascot7) vs. All accession = 1,254 vs. 3,581 at 10 dpa, 876 vs. 1,365 at 20 dpa; Table S2).

We evaluated whether the effects of human selection were biased with respect to the genomic distribution of the effected loci. To do this we tabulated differentially expressed genes by chromosome, and then calculated an expectation based on a null hypothesis of equal distribution, calibrated by the number of genes in each scaffold. This analysis revealed that chromosomes 8 and 1 were differentially targeted during domestication at 10 and 20 dpa, respectively (red text in Table S3). With respect to the latter observation about chromosome 1, the results reflect a putative nuclear mitochondrial DNA (NUMT) sequence block (Figure S4) [16] that contained an unexpectedly high number of up-regulated genes at 20 dpa in both “domestication” (wild vs. domesticated) and “development” (10 vs. 20 dpa) contrasts. For example, during domestication 36 of 84 differentially expressed genes on chromosome 1 were included in this NUMT block and 12 genes are found to be mitochondrial genes, including eight NADH dehydrogenase and four cytochrome-c-related genes.

The comparison of wild and domesticated cottons highlights the fact that the transcriptome of developing cotton fibers was highly altered by five thousand years or more of domestication and crop improvement. To explore this complexity, we first investigated genes previously inferred to be involved in fiber initiation, elongation, and secondary wall biosynthesis (reviewed in [28]). Interestingly, most of the genes involved in the first (initiation) and third (secondary wall biosynthesis) of these stages were up-regulated in wild cottons compared to domesticated cottons at 10 and 20 dpa, respectively (Table S4). In contrast, many genes involved in fiber elongation were highly up-regulated in domesticated cotton compared to wild cottons at 10 dpa, while several genes from this same developmental stage were up-regulated in wild cottons at 20 dpa, encoding annexin, actin depolymerizing factor, FASCICLIN-like arabinogalactan-protein, and tubulin alpha-2 chain (Table S4). Considering expression levels of these genes, differentiation between wild and domesticated cottons was greater during fiber elongation, which is also true at the global transcriptome level, as reported here.

**Figure 2:**
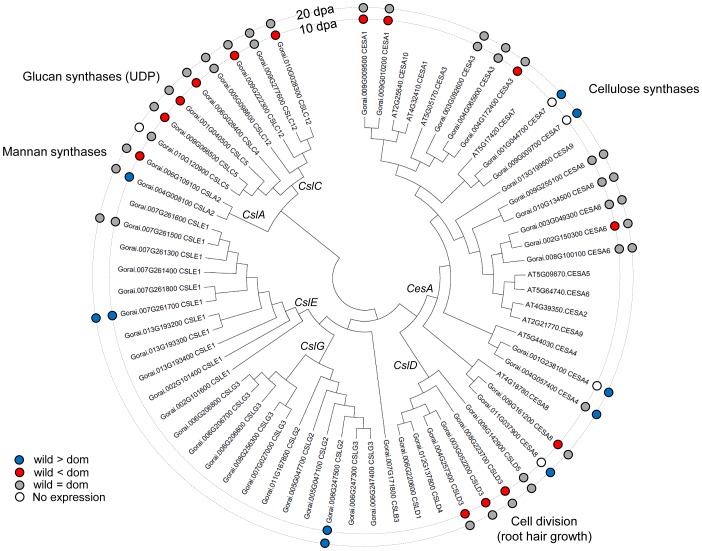
The phylogeny of cellulose synthase (*CesA*) and cellulose synthase-like (*CSL*) genes. Inside and outside circles show differential expression between wild and domesticated cottons at 10 and 20-regulation at domesticated cottons, while blue one indicates up-regulation at wild cottons during domestication. Grey circle shows no differential expression, while white circle designates no expression (zero read count).

Among the 3,581 genes differentially expressed between wild and domesticated cotton at 10 dpa, we tabulated the most highly up-regulated genes in wild or domesticated cottons, relative to their counterparts, with respect to fold change (with RPKM≧50). This analysis reveals that many genes involved in fiber elongation were over-expressed in domesticated cottons, including profilin 1, HXXXD-type acyl-transferase family protein, expansin A8, beta-6 tubulin, FASCICLIN-like arabinogalactan 9 (*FLA9*), and 3-ketoacyl-CoA synthase 2 (*KCS2*) (Table 3). *KCS* genes are involved in fatty acid elongation and are known to be highly expressed during fiber elongation [29]–[31]. In this study, nine and three of 27 *KCS*s were up-regulated in domesticated cotton compared to wild types at 10 and 20 dpa, respectively (Figure S5). Notably, five of nine differentially expressed *KCS*s showed A-homoeolog expression bias, while the other four exhibited no bias (Table S5). *CesA* and *Csl* play critical roles in cell wall biosynthesis [17], [18], [23] and were differentially expressed between wild and domesticated cottons. Five of 18 *CesA*, five of seven *CslC* and three of six *CslD* genes in the reference genome were up-regulated in domesticated cotton at 10 dpa (Figure 2).

**Table 3:** The most abundantly up-regulated genes in domesticated cottons (log2FC>0) or wild cottons (log2FC<0) relative to their counterparts at 10 dpa.

GoraiID	Sequence description	Expression level (RPKM)		log2FC*
		wild	dom	
Gorai.009G028500	Profilin 1	7	755	7.117
Gorai.012G006600	HXXXD-type acyl-transferase family protein	17	292	4.390
Gorai.010G045500	glycerol-3-phosphate acyltransferase 3	4	56	4.014
Gorai.006G240600	Lipid-transfer protein	38	229	2.923
Gorai.002G193500	proline-rich protein 2	383	2260	2.880
Gorai.012G014400	expansin A8	10	52	2.706
Gorai.004G211800	beta-6 tubulin	103	431	2.378
Gorai.006G150100	NDR1/HIN1-like 1	26	102	2.311
Gorai.006G000200	Chalcone and stilbene synthase family protein	29	112	2.264
Gorai.011G035400	GDSL-like Lipase/Acylhydrolase protein	354	1346	2.245
Gorai.002G245900	gamma tonoplast intrinsic protein	246	924	2.230
Gorai.013G177600	glutathione S-transferase TAU 19	14	52	2.174
Gorai.013G270600	heat shock protein 70B	25	89	2.125
Gorai.006G150600	Eukaryotic aspartyl protease family protein	383	1293	2.075
Gorai.008G155400	FASCICLIN-like arabinoogalactan 9	569	1901	2.058
Gorai.011G165200	3-ketoacyl-CoA synthase 2	63	203	2.018
Gorai.002G161400	polygalacturonase 2	18	59	2.009
Gorai.006G148600	syntaxin of plants 121	31	100	2.009
Gorai.010G069000	alpha/beta-Hydrolases superfamily protein	30	97	2.005
Gorai.002G248400.2	plasma membrane intrinsic protein 2	27	88	2.001
**Gorai.008G131700**	O-methyltransferase 1 (OMT1)	365	3	−6.422
**Gorai.007G336600**	Plant invertase/pectin methylesterase inhibitor	134	2	−5.544
**Gorai.007G029700**	HXXXD-type acyl-transferase family protein	213	4	−5.341
**Gorai.008G198200**	Cytochrome P450 superfamily protein (CYP75B1)	239	8	−4.642
Gorai.001G136100	Lipid-transfer protein	57	3	−4.155
**Gorai.013G023400**	Chalcone-flavanone isomerase family protein	709	36	−3.988
Gorai.010G255300	Clathrin light chain protein	62	3	−3.947
**Gorai.011G161300**	Chalcone and stilbene synthase family protein	1541	88	−3.811
**Gorai.004G205900**	leucoanthocyanidin dioxygenase	1744	101	−3.785
**Gorai.008G062900**	flavanone 3-hydroxylase	5324	349	−3.611
**Gorai.011G161200**	Chalcone and stilbene synthase family protein	1120	74	−3.604
**Gorai.004G105500**	NAD(P)-binding Rossmann-fold superfamily protein	443	33	−3.447
Gorai.009G182300	ethylene-forming enzyme	112	9	−3.360
**Gorai.005G035100**	Chalcone and stilbene synthase family protein	1752	143	−3.297
**Gorai.004G184100**	NAD(P)-binding Rossmann-fold superfamily protein	329	29	−3.209
**Gorai.001G134900**	Cytochrome P450 superfamily protein (CYP75B1)	2157	193	−3.166
Gorai.007G322600	metallothionein 3	82	8	−3.133
Gorai.004G277300	Histone superfamily protein	84	8	−3.064

Genes were filtered by RPKM≧50 in either wild or domesticated cottons. Bold indicates genes involved in phenylpropanoid metabolism. Eleven and four genes up-regulated in domesticated and wild cottons, respectively, were removed because of no annotation.

Log2 Fold Change calculated from raw mapped read numbers using DESeq software.

*FLA*s have been classified into four groups [32], but the function of only a few *FLA*s are known. For example, in *Arabidopsis*, *FLA4* or *SOS5* (At3g36550) plays a role in cell expansion [33], and several *FLA* homologues of *G. hirsutum* were highly expressed during fiber elongation [34]. We also observed several *FLA* homologues that were up-regulated in domesticated cottons compared to wild cottons at 10 dpa, including two *SOS5* homologues and three of four *AtFLA7* homologues (Figure S6).

**Figure 3:**
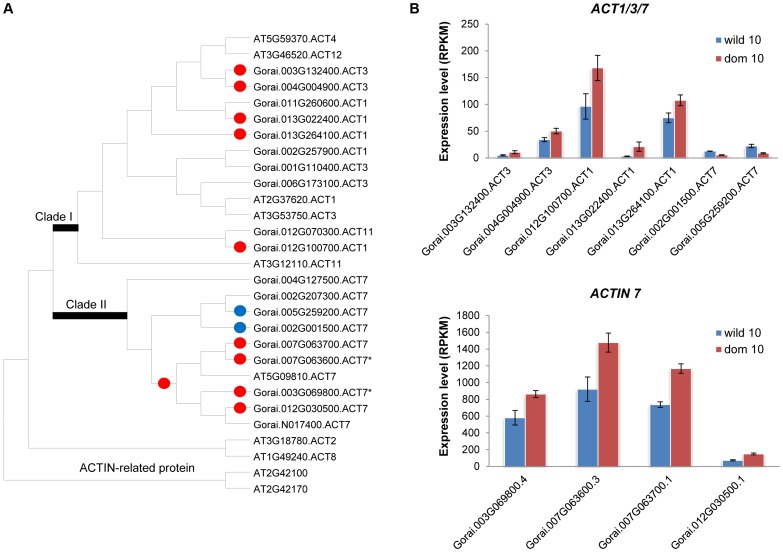
Phylogenetic relationship of *ACTIN* and their gene expression patterns at 10 dpa. (A) Phylogenetic relationship of *G. raimondii ACTIN* genes. Red and blue dots on the node indicate up- or down regulation at domesticated cottons, respectively. Asterisk (*) shows ACTIN homologues previously studied. (B) Expression patterns of differentially expressed *ACT* genes. Bar denotes standard error.

Profilin (*PRF*) and its partners (e.g., actin, tubulin, and villin), which play an important role in actin polymerization [7], [35], were also up-regulated in domesticated cotton, and their expression levels were high, except for villins (Figure S7). Consistent with Bao et al. [7], *PRF1* exhibited the highest expression differences between wild and domesticated cottons, and *PRF3* and *PRF4* were up-regulated in domesticated cottons relative to wild cottons (Figure S7). However, the other two *PRF* genes were not differentially expressed (cf. ref [7]), a conflict perhaps explained by the larger number of accessions studied here. As for *ACTIN* (*ACT*), there were two main clades of *Gossypium ACT*s, *ACT1/3/4/11/12* (clade I) and *ACT7* (clade II) (Figure 3). These two clades are distinct in their gene expression patterns; members of *ACT1/3/4/11/12* generally are expressed in reproductive organs, such as pollen, pollen tubes, and ovules of *Arabidopsis*, while *ACT7* is expressed in vegetative tissues, including root hairs and trichomes along with *ACT2* and *ACT8* [36]–[39]. In the present study, at 10 dpa nine and two *ACT* genes were up-regulated in domesticated and wild cottons, respectively (Figure 3). Interestingly, all of the genes closely related to *Arabidopsis ACT7* were commonly and highly up-regulated in domesticated cottons, except Gorai.N017400. Previously identified *GhACT1*, which was shown to participate in fiber elongation [40], showed the highest similarity to Gorai.007G063600 (probably *GhACT2*; see Table S4) included in the *ACT7* clade. Notably, Gorai.007G063600 seems to be duplicated; its duplicate, Gorai.007G063700, was also up-regulated in domesticated cottons with expression levels similar to that of Gorai.007G063600 (Figure 3). Other *ACT* genes, which are members of clade I or which are up-regulated in wild cottons, exhibited relatively low expression levels compared to *ACT7* homologues.

**Figure 4:**
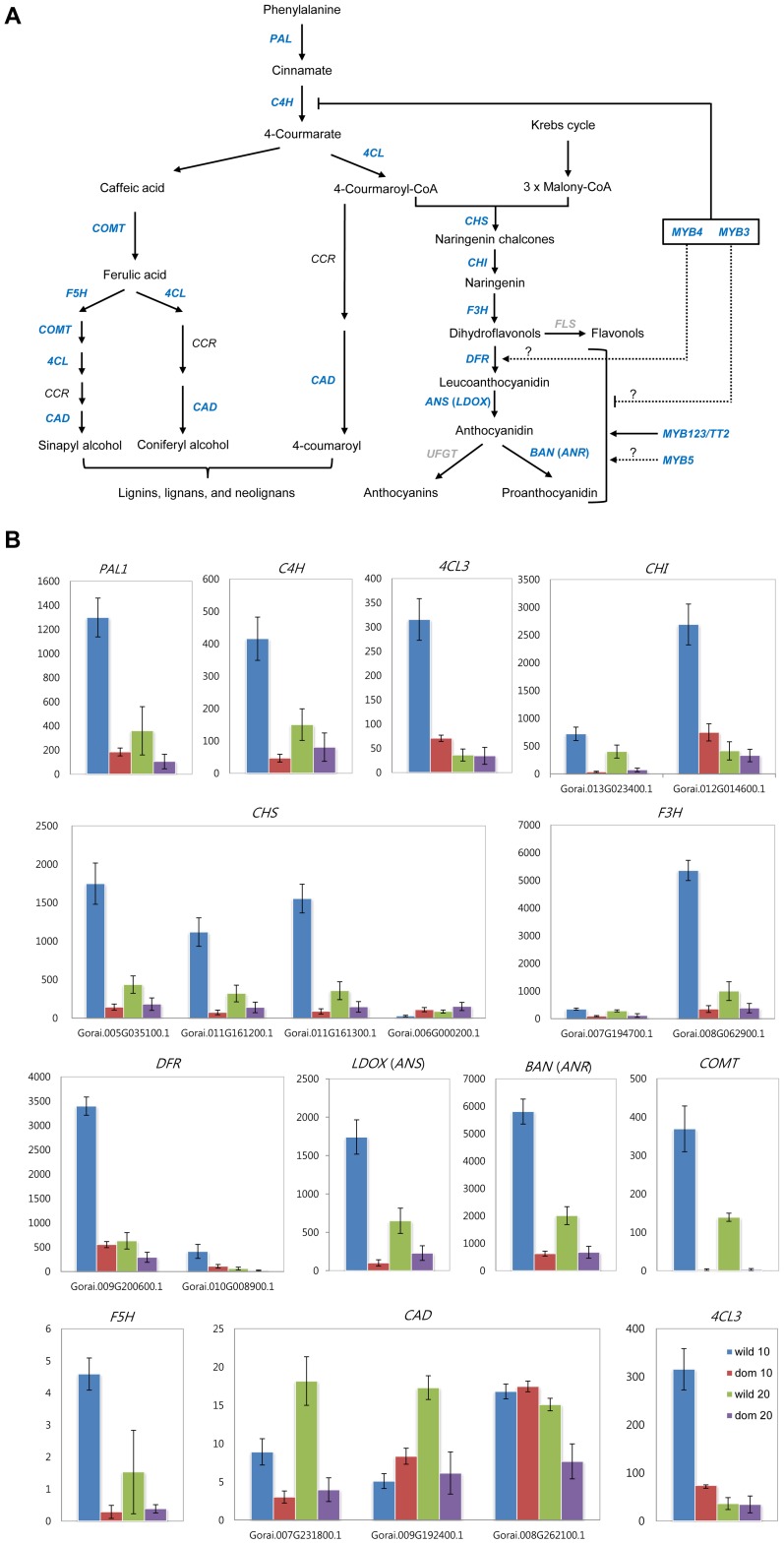
Expression patterns of genes related to phenylpropanoid pathway (A) and their actual expression levels (B) in wild and domesticated cottons. Blue text in (A) indicates up-regulation in wild cottons relative to domesticated cottons at 10 dpa, and bar in (B) denotes standard error. PAL, phenylalanine ammonium lyase; C4H, cinnamate-4-hydroxylase; 4CL, 4-coumaroyl-CoA synthase; CHI, chalcone isomerase; F3H, flavonol 3-hydroxylase; FLS, flavonol synthase; DFR, dihydroflavonol-4-reductase; ANS, anthocyanin synthase, LDOX, leucoanthocyanidin dioxygenase; ANR, anthocyanidin reductase; COMT, F5H, CCR, CCD.

**Figure 5:**
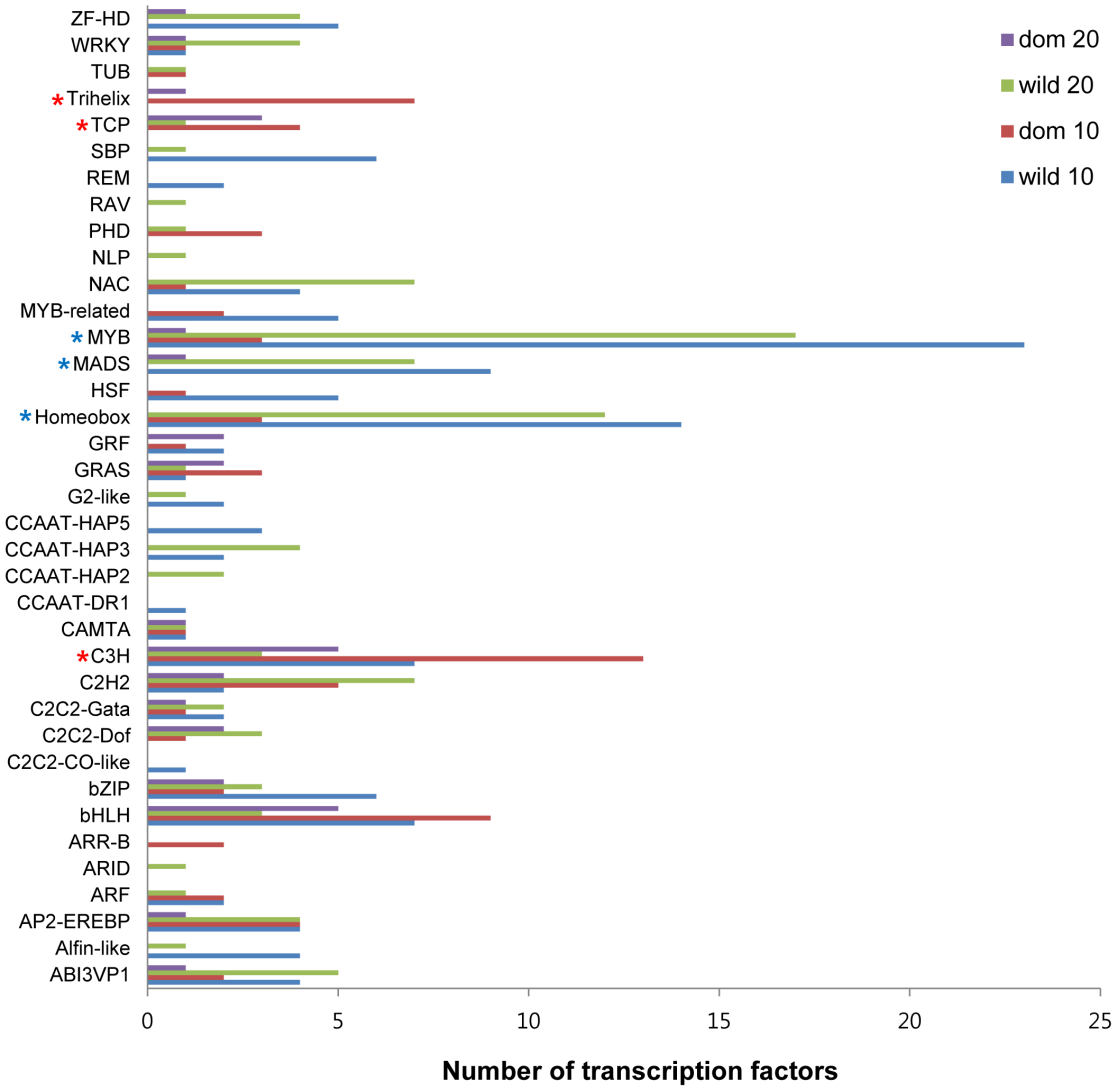
Number of TFs differentially expressed between wild and domesticated cotton. Blue and red asterisk (*) represent over-represented TFs in wild and domesticated cottons relative to their counterparts at both developmental time points, respectively (proportion test; P<0.05).

In contrast to some key genes observed to be up-regulated under domestication, in wild cottons some genes involved in phenylpropanoid metabolism, such as flavonoid biosynthesis and anthocyanin biosynthesis, were highly up-regulated at both developmental time points compared to their counterparts in domesticated cottons (Figure 4). For example, *PHENYLALANINE AMMONIA LYASE 1* (*PAL1*) exhibited 5.6 times higher expression than in domesticated cottons at 10 dpa, and other genes involved in this pathway showed similar patterns (Figure 4B). In addition, some MYB transcription factors (TFs) were also up-regulated in wild cotton compared to domesticated cotton (Figure 5, Figure S8). In particular, half of the 23 differentially expressed MYB TFs in wild cottons were related to the phenylpropanoid pathway, as noted earlier [16], and their up-regulation was observed at both developmental stages (Figure S8).

At 20 dpa, similar sets of genes were differentially expressed, but their expression levels were relatively lower compared to those observed in 10 dpa (e.g., Figure S3, S4, S5, S6, S7, S8). Many of the genes up-regulated in domesticated cottons were related to protein synthesis (see below) or were found in a putative nuclear mitochondrial DNA (NUMT) sequence block (Table S3; see above). In addition and importantly, 14–19% of the differentially expressed genes encode unknown proteins, in agreement with previous reports [13], [21]; these genes become obvious targets for future functional analysis, to discover their roles in cellular development and in evolution.

Among the 2,830 TFs that are annotated in the cotton genome, fewer than 10% were differentially expressed between wild and domesticated cottons. Specifically, 266 (184 vs. 82 up-regulated in wild vs. domesticated, or the reverse) and 132 (100 vs. 32 up-regulated in wild vs. domesticated, or the reverse) were differentially expressed at 10 and 20 dpa, respectively. Among these, only 48 TFs were expressed at the level of RPKM≧50, indicating that the majority of TFs are not highly expressed in fibers. Of these 48 highly expressed TFs, 27 and 16 were up-regulated in wild and domesticated cottons relative to their counterparts, respectively. Five genes were not differentially expressed including *GLABRA2* (*GL2*) and *MYB60* which have been functionally studied. *GL2* regulates cell wall-related gene expression (*CeSA5* and *XTH17*) during root development in *Arabidopsis* [41], while *MYB60* is involved in stomatal regulation and root growth under drought stress in grapevine [42] and *Arabidopsis* [43], or repressing anthocyanin biosynthesis in lettuce [44]. As for TFs up-regulated in wild cottons relative to domesticated cottons, three TFs families were the most commonly represented, including homeobox, MADS and MYB TFs (Figure 5, Table S6). This is consistent with previous studies on the importance of MYBs in fiber development [45]–[48], but over-representation of MADS genes has not previously been reported for cotton fibers. MADS genes that were differentially expressed were related to carpel (e.g., *AGAMOUS*, *SHATTERPROOF1*, *SEPALLATA*) and seed development (*SEEDSTICK*); results here suggest the possibility that these genes have found a new role in fiber development. In domesticated cottons, three TFs, C3H, TCP, and trihelix, were the most commonly represented classes among the 48 TFs (Figure 5, Table S6); this includes a TF that recently has been identified as important for fiber development in both *G. barbadense* [49] and *G. hirsutum* [50]. Gorai.007G036800, a homologue of *GhTCP14* [50], was up-regulated in domesticated cottons relative to wild cottons, supporting its relatedness to fiber elongation.

### Functional analyses of differential expression

To evaluate whether specific biological processes were enriched in representation by either development or domestication, two different functional analyses were performed, the Singular Enrichment Analysis (SEA) and the Parametric Analysis of Gene set Enrichment (PAGE). Although SEA and PAGE deploy different strategies, both methods yielded similar results. Thus, we present only SEA results here (Table S7), to highlight some of the differences between wild and domesticated cottons. In general, during development more biological processes were differentially regulated in wild cottons than domesticated cottons (wild vs. domesticated = 71 vs. 1 biological processes (P) in Table S7A), as expected based on the degree of differential expression found in comparison of two developmental time points in wild and domesticated cottons (Figure 1). For example, at 10 dpa in wild cottons, genes related to lipid metabolism were enriched, including fatty acid biosynthetic process, very-long-chain fatty acid (VLCFA) metabolic process, sterol biosynthetic process, and steroid biosynthetic process, and secondary metabolites biosynthesis process was also up-regulated, including phenylpropanoid, coumarin, flavonoid, and anthocyanin biosynthesis processes (Table S7A). In addition, gibberellic acid (GA) mediated signaling pathway was also over-represented at 10 dpa in wild cottons, noting that GA is required for fiber initiation and elongation [51], [52]. At 20 dpa in wild cottons, in addition to in cell wall organization or biogenesis, genes involved in response to abiotic and biotic stimuli, such as water deprivation, organic substance, chemical and hormone stimuli were over-represented relative to 10 dpa (Table S7A). In domesticated cottons, there was no biological process enriched between 10 and 20 dpa based on PAGE analysis (data not shown), while SEA results indicated that genes related to lipid metabolic process were up-regulated at 10 dpa compared to 20 dpa (Table S7A).

In the domestication contrast, SEA results showed that more biological processes were up-regulated in domesticated cottons than in wild cottons (123 in domesticated cottons vs. 49 in wild cottons; Table S7B). Many up-regulated genes in wild cottons relative to domesticated cottons at 10 dpa were related to protein-DNA complex assembly, nucleosome assembly, response to disaccharide stimulus, and secondary metabolite synthetic processes, such as anthocyanin, flavonoid, and phenylpropanoids (Table S7B). Consistent with this result, cellular components, such as chromosome and nucleosome, and molecular function of transcription factor activity were highly enriched in wild cottons (Table S7B). At 20 dpa of wild cottons, genes involved in cell wall macromolecule metabolism and amine catabolism were up-regulated relative to domesticated cottons, suggesting that secondary cell wall synthesis is active in wild cottons. Amine catabolism involves protein degradation, which may generate nitrogen-containing compounds for secondary metabolite synthesis. In fact, most genes related to amine catabolism were associated with phenylpropanoid biosynthesis, for example, 4-coumarate:CoA ligase 1 (*4CL1*), *PAL1*, cinnamyl alcohol dehydrogenase (*CAD*), and cinnamoyl CoA reductase 1 (*CCR1*). On the other hand, domesticated cottons were defined by fiber elongation-related processes (e.g., vesicle-mediated transport, actin cytoskeleton organization, and cellulose metabolism) at 10 dpa and energy generation and protein synthesis at 20 dpa (Table S7B). For example, many genes differentially expressed at 20 dpa of domesticated cottons compared to wild cottons were involved in RNA elongation, cellular respiration along with oxidative phosphorylation, and protein synthesis (translation). Also, some genes involved in fatty acid biosynthesis were up-regulated, perhaps to facilitate membrane growth and turnover during fiber elongation and maturation [53].

### Homoeolog-specific biases and change during development and domestication

To explore whether there is a bias in usage of parental gene copies (homoeologs) during development and domestication, a phenomenon termed homoeolog expression bias [54], [55], the relative contribution of homoeologs to total gene expression was investigated. An average of 17,800 genes had homoeolog-specific reads, of which 17.5 to 53.5% showed unequal (biased) expression in any one case (Table 4). Notably, by far the highest percentage of genes showing biased homoeolog contributions to the transcriptome was at 10 dpa for domesticated cottons, a rate nearly twice that observed at 20 dpa. In addition, more genes at 10 dpa than at 20 dpa exhibit homoeolog bias in all comparisons (Table 4). Considering the entire data sets, which include all genes having a minimum number of homoeolog-specific reads (RPKM≧1), there is no global bias in homoeolog expression in either wild or domesticated cottons; that is, despite appreciable gene-level bias, the number of genes that exhibit either A_t_ or D_t_ bias (where the lower case t designates homoeolog in the allopolyploid) are approximately equal (balanced homoeolog bias, sensu Grover et al. [54]). This same result also characterizes most other comparisons.

**Table 4:** Homoeolog-specific bias in developing cotton fiber.

	Total # genes^a^	A_t_ = D_t_	A_t_>D_t_	A_t_<D_t_	Total biased genes (%)
Entire data					
wild 10 dpa	18,315	13,815	2,258	2,242	4,500 (24.6%)
wild 20 dpa	19,163	14,941	2,141	2,081	4,222 (22.0%)
dom 10 dpa	17,551	11,641	2,966	2,944	5,910 (33.7%)
dom 20 dpa	15,901	13,126	1,382	1,393	2,775 (17.5%)
DE genes during development					
wild 10 dpa	2,315	1,335	468	512	980 (42.3%)
wild 20 dpa	2,612	1,632	490	490	980 (37.5%)
dom 10 dpa	626	291	163	172	335 (53.5%)
dom 20 dpa	693	422	131	140	271 (39.1%)
DE genes during domestication					
wild 10 dpa	1,691	1,256	223	212	435 (25.7%)
dom 10 dpa	1,605	1,066	289	250	539 (33.6%)
wild 20 dpa	951	567	183	201	384 (40.4%)
dom 20 dpa	631	360	131	140	271 (42.9%)

A_t_ = A-homoeolog; D_t_ = D-homoeolog; A_t_ = D_t_, equal expression of homoeologs; A_t_>D_t_, biased expression of the A_t_ homoeolog; A_t_<D_t_, biased expression of the D_t_ homoeolog; DE = differential expression from the contrasts of development (10 vs. 20 dpa) or domestication (wild vs. dom).

^a^ includes genes having at least RPKM≧1 in either A or D-homoeolog specific reads across all biological replicates.

To assess how homoeolog usage is affected during development and by domestication, we compared the *same* homoeolog from two different developmental stages or from the two pools of wild vs. domestication cottons. During cotton fiber development from 10 dpa to 20 dpa, we observed more homoeolog expression change in wild cottons than in domesticated cottons (4,358 of 22,012 (19.8%) in wild vs. 2,110 of 19,974 (10.6%) in domesticated; Table 5) although there is no difference in DE genes between wild and domesticated cottons (Table 5). There were more homoeolog changes at 10 dpa than at 20 dpa (3,350 of 20,994 (16.0%) at 10 dpa vs. 2,433 of 21,230 (11.5%) at 20 dpa; Table 5) as a result of domestication. However, we observed balance in most comparisons; for example, there are similar numbers of A_t_ and D_t_ down- or up-regulation during fiber development in wild cottons (down-regulation of A_t_ vs. D_t_ = 290 vs. 321, up-regulation of A_t_ vs. D_t_ = 320 vs. 336; Table 5). When combined with the results from Table 4 (described above), we infer that homoeolog modulations (both expression and change) were balanced in cotton fiber regardless of development and domestication.

**Table 5:** Homoeolog expression changes across two developmental stages and during domestication.

Comparison	Homoeolog changes from 10 dpa to 20 dpa	Gene pairs in entire data				Gene pairs in DE genes			
		wild cotton	%	dom. cotton	%	wild cotton	%	dom. cotton	%
Development	No changes	17,654	80.2	17,864	89.4	306	9.5	86	9.1
	Total changed	4,358	19.8	2,110	10.6	2,929	90.5	861	90.9
	both down-regulated	766	3.5	246	1.2	692	21.4	142	15.0
	A_t_ down-regulated	615	2.8	302	1.5	290	9.0	76	8.0
	D_t_ down-regulated	603	2.7	283	1.4	321	9.9	79	8.3
	both up-regulated	1,045	4.7	389	1.9	966	29.9	273	28.8
	A_t_ up-regulated	694	3.2	478*	2.4	320	9.9	150	15.8
	D_t_ up-regulated	622	2.8	411	2.1	336	10.4	141	14.9
	A_t_ up, D_t_ down	7	0.0	0	0.0	4	0.1	0	0.0
	A_t_ down, D_t_ up	6	0.0	1	0.0	0	0.0	0	0.0

	Homoeolog changes from wild to dom	Gene pairs in entire data				Gene pairs in DE genes			
		10 dpa	%	20 dpa	%	10 dpa	%	20 dpa	%
Domestication	No changes	17,644	84.0	18,797	88.5	1,037	31.9	178	15.8
	Total changed	3,350	16.0	2,433	11.5	2,215	68.1	948	84.2
	both down-regulated	480	2.3	337	1.6	464	14.3	250	22.2
	A_t_ down-regulated	620	3.0	456	2.1	290	8.9	118	10.5
	D_t_ down-regulated	637	3.0	494	2.3	355*	10.9	152	13.5
	both up-regulated	449	2.1	275	1.3	409	12.6	158	14.0
	A_t_ up-regulated	585	2.8	440	2.1	348	10.7	145	12.9
	D_t_ up-regulated	554	2.6	416	2.0	341	10.5	124	11.0
	A_t_ up, D_t_ down	16	0.1	8	0.0	6	0.2	0	0.0
	A_t_ down, D_t_ up	9	0.0	7	0.0	2	0.1	1	0.1

indicates that the number of genes belonging to “A_t_ up-regulated” and “D_t_ down-regulated” is statistically different from “D_t_ up-regulated” and “A_t_ down-regulated”, respectively (P<0.05).

## Discussion

### The complex cotton fiber transcriptome

Cotton fiber development involves an extraordinarily complex biology regulated by multiple and diverse pathways and transcriptional regulatory networks. In this study, we generated global transcriptome profiles of developing cotton fibers from multiple accessions of wild and domesticated *G. hirsutum*. Using RNA-Seq, we determined that at least one-third and likely about half of the genes (depending on the RPKM threshold) in the cotton genome are expressed in developing fibers. This number is consistent with previous estimates of the fiber transcriptome diversity generated for *G. hirsutum* cv. TM1 and *G. arboreum*, notwithstanding the technical differences among studies [20], [21]. It is striking that the genic diversity in the transcriptome of fibers, which are single cells, is comparable to that of entire young leaves of *G. hirsutum* (Table 2) [55], which are far more complex organs comprising multiple different cell types and with varying cellular specializations and diverse metabolic roles. This comparison justifies the perspective that the cotton fiber transcriptome is extraordinarily rich and that it is subject to complex transcriptional regulation during fiber development.

One of the justifications for the experimental design used in the present study was to attempt to account for expression variation that might occur *within* wild and *within* domesticated *G. hirsutum* and hence account for this variation to strengthen inferences about the differences *between* these groups. Accordingly, we selected multiple accessions within each pool. For the two developmental stages studied here, 10 and 20 dpa, we estimate that, respectively, 3.1% (1,144) and 9.3% (3,487) of the duplicate gene pairs in the tetraploid cotton genome were differentially expressed between 10 and 20 dpa in domesticated and wild cottons, respectively. Importantly, when we reanalyze our RNA-Seq data, restricting our attention to the same two accessions as used in Rapp et al. [13], TM1 (domesticated) and TX2094 (wild), we observe about a 40% increase in the number of differentially expressed genes (6,908 vs. 4,946) (Table S2). These data indicate that inclusion of multiple accessions narrowed the differences between the two pools “wild” and “domesticated”, boosting confidence in inferences regarding the effects of human selection, and in the process identifying expression variation arising from other causes.

In addition to bolstering the notion that the fiber transcriptome is highly diverse and dynamic, the results presented here include deep and rich data sets that can be mined for clues regarding processes of cellular development and those that have been most strongly affected by human-mediated directional selection under domestication. The data also provide new information on homoeolog usage and biases in a polyploid cell type. Each of these topics is discussed in more detail in the following.

### Domestication prolonged fiber elongation during development

A body of work has established that cotton fiber development consists of stages that are well-defined temporally, i.e., fiber initiation (0–3 dpa), primary cell wall synthesis and elongation (3–15 dpa), transition to secondary cell wall growth (15–20 dpa), secondary wall biosynthesis (20–40 dpa), and maturation (40–50 dpa) [18]. Our observation of relatively little differentiation between 10 and 20 dpa in domesticated cottons suggests that the fiber primary elongation developmental program had continued to 20 dpa, consistent with a previous study based on fiber growth curves [56]. In domesticated cotton, the rates of fiber growth and maturation were the highest between 10 and 15 dpa, but extended up to 30 dpa (20 days of fiber elongation), while fibers of wild cottons elongated fastest between 15 and 20 dpa (5 days of elongation) [56], [57]. In particular, cotton fiber from *wild G. hirsutum* already reached >90% of maturation around 20 dpa, indicating early termination of fiber elongation, and likely entry into the transition phase leading to secondary wall synthesis. Our transcriptome profiling results showed that gene expression patterns were significantly more differentiated between 10 and 20 dpa in wild cottons, perhaps reflecting this subtle temporal shift in the fiber developmental program. Based on fiber growth curve analyses, fiber elongation appears modest until 15 dpa in wild cottons, yet is almost complete by 20 dpa [56]. Thus, 10 dpa from wild cottons represents an early stage of fiber elongation in wild, relative to domesticated, *G. hirsutum*, while by 20 dpa fibers from wild cotton likely have completed primary cell wall synthesis and have entered the transition to secondary wall synthesis. This inference is also supported by expression patterns of genes previously reported from gene-by-gene surveys; wild cottons showed more up-regulations of genes related to fiber initiation and secondary wall biosynthesis at 10 and 20 dpa, respectively, compared to domesticated cottons (Table S4). This difference in developmental timing might account for the three-fold increase in the number of differential expressed genes between the two developmental stages of wild cottons relative to domesticated cottons (Figure 1).

Previous studies support this interpretation of a period of prolonged fiber elongation under domestication, and in parallel in different domesticated cotton species. Notably, similar conclusions have been reached for diploid domesticated cotton, *G. arboreum* [58], and in a second domesticated allopolyploid cotton, *G. barbadense* [59]. In addition, Hu et al. showed, in a recent, high-throughput iTRAQ proteomic analysis, that the proteome of domesticated cotton during early fiber elongation (5–10 dpa) was similar to that of later developmental stage of wild cottons (10–20 dpa) [59]. Thus, it seems that human domestication may have induced parallel prolongations and developmental shifts on the fiber elongation period in both diploid and allopolyploid species, as evidenced by growth curve analysis [56], and both transcriptomic and proteomic analyses [58], [59]. These studies, as well as light microscopy observations [13] which demonstrate that wild and domesticated *G. hirsutum* share similar timing and morphology of early wall thickening, point to the need to develop a deeper understanding of the underlying developmental programs and architecture of fiber growth and evolution. In a recent metabolic profiling study [12] of a lintless mutant and its wild type (WT) *G. hirsutum* relative, 487 metabolites identified from nine developmental time points clearly differentiated the metabolomic profiles of the lintless mutant from that of WT cotton during elongation, but there was no clear differentiation between the two forms during fiber initiation (−3 to 3 dpa). This suggests that the short period of fiber elongation in the lintless mutant, where fiber cells become arrested at about 6 mm of linear growth, resembles, to a certain extent, the wild representatives of the domesticated species. Considering the evolutionary, morphological transformation of fiber from lintless in wild to linted in domesticated cottons [4], prolonged fiber elongation was a key innovation for longer fiber, which is apparent at the transcript, protein, and metabolite levels. Additional insight into the nature of this developmental shift will probably arise from further integrated studies of various “omics”, combined with a denser sampling of developmental time points.

Analysis of differential expression showed that wild cottons deployed a higher number of biological processes compared to domesticated cottons, for example SEA results showed there were 77 vs. 1 in wild vs. domesticated cottons, respectively (Table S7A). In particular, as consistent with little differentiation between 10 and 20 dpa in domesticated cottons, only one biological pathway, lipid metabolic process, was over-represented at 10 dpa relative to 20 dpa. However, in wild cottons, different metabolic pathways were over-represented in the DE gene sets that characterize development, including fatty acid biosynthesis and secondary metabolite biosynthesis at 10 dpa and cell wall organization and biogenesis at 20 dpa (Table S7A). Interestingly, the GA mediated signaling pathway was enriched at 10 dpa in wild cottons. Considering that GA is required for fiber growth [52] and shows the highest level in 10 dpa fibers [51], up-regulation of this pathway indicates active fiber elongation at 10 dpa compared to 20 dpa in wild cottons. For example, *GAST1 PROTEIN HOMOLOG 4* (*GASA4*) was known to promote GA response and regulate redox status in *Arabidopsis* [60], and two cotton homologues (Gorai.006G017000, Gorai.012G054200) were highly expressed and up-regulated at 10 dpa compared to 20 dpa in wild cottons. On the other hand, “response to stress” pathways were enriched in 20 dpa wild cottons, thus, genes related to stress were up-regulated, including those in the dehydrin family protein (*EARLY RESPONSIVE TO DEHYDRATION 10*; Gorai.002G119600), senescence-associated genes (Gorai.012G124700), late embryogenesis abundant like 5 (Gorai.002G119600), and cold-regulated 47 (Gorai.009G189500). These genes are known to be expressed in response to abiotic stress, such as high salinity, drought, and cold in *Arabidopsis* [61]–[63], implying that wild cottons are utilizing stress-response pathways at 20 dpa. This inference was also supported by up-regulation of many ROS genes (see below), suggesting that up-regulation of stress-related gene expression in wild cottons could have resulted in halting fiber elongation and promoting the transition to secondary wall biosynthesis.

Many genes related to phenylpropanoid biosynthesis were up-regulated during fiber development in wild cottons (Figure 4A) with expression levels that were significantly higher at 10 than at 20 dpa (Figure 4B), in agreement with previous studies [64], [65]. The same differential regulation characterized wild vs. domesticated cottons at both developmental time points (discussed further below).

Wild and domesticated cottons exhibited no differences in homoeolog utilization at both expression (Table 4) and change (Table 5). This is consistent with Yoo et al. [55] and Rambani et al. [66] which showed an overall equal usage of both homoeologs in young leaf and petal tissues of both TX2094 and Maxxa, respectively. These results suggest that domestication has not affected the utilization of homoeologs from the two co-resident genomes of allopolyploid cotton. However, further study is required to evaluate whether this equal usage of homoeologs was derived from vertical inheritance of progenitor A and D genome conditions, of if instead *trans*-acting regulatory factors overwhelmed pre-existing, evolved *cis*- and *trans*- differences that accumulated during evolutionary divergence of the two progenitor diploids

### Domestication may have reallocated resources from stress-response pathways to fiber growth

Over the course of several thousands of years of domestication and selection, the short, coarse, and brown fibers of wild *G. hirsutum* were transformed into the long, strong, and fine white fibers that characterize modern upland cultivars. Recent large-scale transcriptomic and proteomics analyses have begun to reveal some of the molecular underpinnings of this remarkable morphological modification [12], [13], [20]–[22], [58], [59], [67]. Here we tried to build on this initial insight into the effects of the domestication and plant improvement process by generating transcriptomes from multiple accessions, thus permitting gene expression changes resulting from domestication to be isolated from those arising from other causes. The wild and domesticated cottons selected exhibit the typical fiber characteristics of their respective pools in fiber color and fiber length (Figure 1), and they also were highly differentiated with respect to their transcriptomes (Figure S1). One key result is the observation of greater gene expression differentiation between wild and domesticated cottons at 10 than 20 dpa (Figure 1), consistent with previous studies [11], [13]. These were partitioned almost equally toward either wild or domesticated cottons (Figure 1; dom vs. wild = 1,839 vs. 1,736). However, if we consider only the more highly expressed genes, i.e., those with a RPKM≧5, twice as many genes were up-regulated in domesticated cottons compared to wild cottons at 10 dpa (dom vs. wild = 1,476 vs. 733), implicating a ramping up of cellular machinery involved in primary wall synthesis and perhaps down regulation of other pathways (see below). A corollary implication is that the majority of up-regulated genes in the wild cottons compared to domesticated cottons at 10 dpa were expressed at a lower level (RPKM<5). Similar trends were observed in the 20 dpa comparison, but with less imbalance and smaller absolute numbers (dom vs. wild = 349 vs. 302 with RPKM≧5).

**Figure 6:**
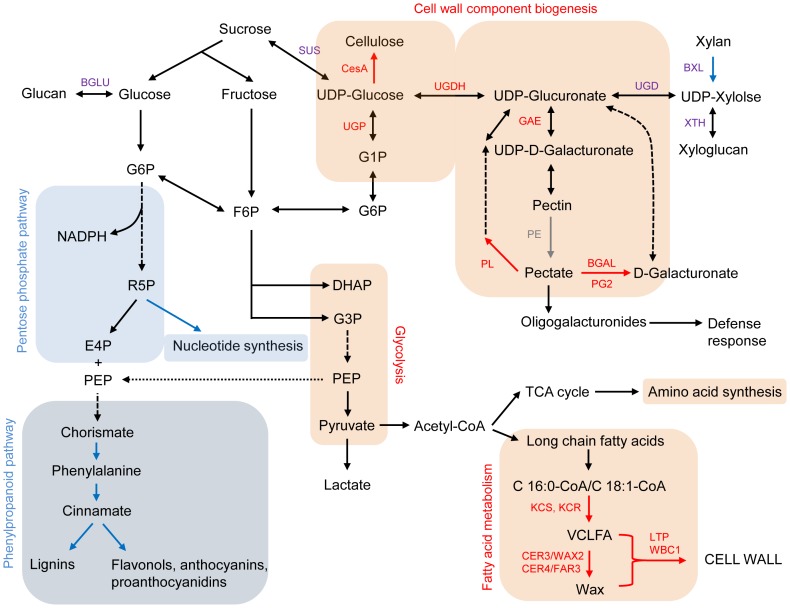
Carbohydrate and fatty acid metabolisms, focusing on cell wall biosynthesis [68]–[70]. Red text and line indicate up-regulated genes or pathways in domesticated cottons relative to wild cottons at 10 dpa, while blue text and lines show up-regulated genes or pathways in wild cottons compared to domesticated cottons at 10 dpa. Purple text indicates that the genes were up- or down-regulated during domestication. Blue and pink shaded boxes show enriched pathways in wild and domesticated cottons compared to their counterparts, respectively. ADPG, ADP-Glucose (ADPG); BGAL, β-galactosidase; BGLU, β-1,3-glucosidase; BXL, β-xylosidase; CER3/WAX2, fatty acid hydroxylase superfamily; CER4/FAR3, fatty acid reductase 3; DAHP, 3-deoxy-D-arabino-heptulosonate-7-phosphate; DHAP, dihydroxyacetone phosphate; E4P, erythrose-4-phosphate; F6P, Fructose-6-Phosphate; GAE, UDP-D-glucuronate-4-epimerase; G1P, Glucose-1-Phosphate; G3P, Glyceraldehyde-3-phosphate; G6P, Glucose-6-Phosphate; KCR, beta-ketoacyl reductase; KCS, 3-ketoacyl-CoA synthase; LTP, lipid transfer protein; PE, pectinesterase; PEP, phosphoenolpyruvate; PG2, polygalacturonase 2; PHS2, alpha-glucan phosphorylase 2; PL, Pectate lyase; R5P, Ribose-5-phosphate; SUS, sucrose synthase; UGD, UDP-glucuronate decarboxylase; UGDH, UDP-glucose-6-dehydrogenase; UGP, UDP-glucose pyrophosphorylase; VLCFA, very long chain fatty acids; WBC1, ATP-binding cassette transporter white-brown complex homolog protein 1; XTH, Xyloglucan endotransglycosylase.

Gene enrichment analyses indicate that specific biological processes were enriched as a consequence of domestication. In particular, the combined significance of functional suggestions become apparent when one considers carbohydrate and fatty acid metabolism with respect to glycolysis, cell wall component biosynthesis, the pentose phosphate pathway, and phenylpropanoid biosynthesis [68]–[70]. In domesticated cottons, carbon resources appear to be more heavily invested in cell wall component biosynthesis, such as cellulose and matrix polysaccharides, as well as energy production through glycolysis (Figure 6). In addition, acetyl-CoA, a product of glycolysis, is linked to synthesis of VLCFAs that are precursors for phospholipids and sphingolipids, essential components of plasma membranes [70]. VLCFAs accumulate preferentially in elongating fibers and *KCS*s, the rate-limiting enzyme in biosynthesis of VCLFAs [71], are also up-regulated during fiber elongation [29]–[31]. In this study, we also observed several *KCS*s that were highly expressed in domesticated cottons at 10 dpa (Figure S5). Notably, five of nine *KCS*s differentially expressed exhibited A-homoeolog expression bias (Table S5), implying that domestication process could have selected maternal parental copy only. Further study on the genome scale is required to elucidate whether this phenomenon is stochastic or linked to specific pathway(s). Other genes related to this pathway were also up-regulated in domesticated cotton compared to wild cottons, including beta-ketoacyl reductase (*KCR*), fatty acid hydroxylase (*CER3*/*WAX2*), fatty acid reductase (*CER4*/*FAR3*), lipid transport protein (*LTP*), and ATP-binding cassette transporter (*WBC1*) (Figure 6). Noteworthy, *CesA*, *CslC* and *CslD* genes were up-regulated in domesticated cottons at 10 dpa only, while *CesA* genes were up-regulated in wild cottons at 20 dpa (Figure 2). This indicates that *CslC* and *CslD* genes have become up-regulated by domestication early in fiber development. Other fiber elongation-related genes, e.g., profilin and its partners, were also up-regulated in domesticated cottons at 10 dpa (Figure 3, Figure S7), including members of one sub-clade of *ACT7* (Figure 3). In *Arabidopsis*, *ACT7* is a vegetative actin, along with *ACT2* and *ACT8*, (the latter two have no obvious homologs in cotton), and are involved in root growth and epidermal cell specification [39]. Here, we observe two sub-clades of *ACT7* in cotton, one up-regulated in wild cottons, while the other is up-regulated in domesticated cottons (Figure 3). Thus, the domestication process may have recruited enhanced utilization of one sub-clade of *ACT7* for greater fiber elongation.

In wild cottons, nucleotide biosynthesis and phenylpropanoid biosynthesis were enriched, based on differential expression. In particular, many genes related to phenylpropanoid biosynthesis were up-regulated during fiber development and domestication in wild cottons (Figure 4A), and their expression levels were much higher at 10 than 20 dpa (Figure 4B), in agreement with previous studies [64], [65]. Notably, phenylpropanoids, particularly flavonoids, are known to inhibit fiber elongation [64], but protect cells from abiotic and biotic stresses [72]. The involvement of flavonoids in fiber processes has been shown in many studies at the transcript, protein, and metabolic levels [12], [13], [20], [64], [73]. Tan et al. [64], in particular, showed that the flavonoid naringenin negatively regulates fiber development and that higher levels of naringenin accumulate in short, brown fibers.

This up-regulation of genes related to phenylpropanoid biosynthesis as well as nucleotide biosynthesis is illustrated in the model suggested in Figure 6, presented within the conceptual framework of carbon/nitrogen balance. For optimal growth and development, carbon and nitrogen metabolism need to be tightly coordinated [74]. Based on the presumed function of the differentially expressed genes, more C compound related pathways are enriched in domesticated cottons relative to wild cottons, as reflected in the greater allocation to cell wall component synthesis (e.g., cellulose, VCLFA), energy generation through glycolysis, and amino acid synthesis (Figure 6). In turn, these biological processes might lower C/N, giving rise to less accumulation of anthocyanin. In contrast, nitrogen related pathways were enriched in wild cottons relative to domesticated cotton, as represented by nucleotide biosynthesis and phenylpropanoid biosynthesis. These two pathways can redirect carbon flow to nitrogen metabolism by diverting glucose-6-phosphate (G6P) into the pentose phosphate pathway or phosphoenolpyruvate (PEP) to the phenylpropanoid pathway (Figure 6). It may be, therefore, that the domestication process reallocated carbon resources toward carbohydrate and fatty acid metabolism. This speculation is also supported by a comparative metabolomics survey [12] of a lintless mutant and its wild type progenitor; the lintless mutant exhibited up-regulation of genes related to nitrogen compound metabolism along with accumulation of nitrogen compounds, compared to its WT. This phenomenon remains to be demonstrated at the metabolic level in wild and domesticated cottons.

Perhaps related to the above are differences in the deployment of stress-response pathways. For example, *GAST1 protein homolog 1* (*GASA1*; Gorai.010G004400), involved in diverse developmental programs and stress responses [75], was highly up-regulated in wild cottons compared to domesticated cottons at 20 dpa. *GASA* genes have been reported to promote cell elongation in petunia flower [76], [77] or arrest cell elongation in gerbera [78] and strawberry [79]. Possibly, up-regulation of *GASA1* in wild cottons at 20 dpa implies a negative regulation of cotton fiber elongation and/or modulation of stress response. Analyses of 176 genes related to the reactive oxygen species (ROS)-scavenging network [80] support the possibility of greater ROS sensitivity of wild cottons at 20 dpa; for example, during development 27 ROS genes were differentially expressed in wild cottons (7 vs. 20 genes = 10 vs. 20 dpa), while there were only 8 ROS genes identified in domesticated cottons, and also more ROS genes were up-regulated in wild than in domesticated cottons. ROS plays different roles depending on concentrations and context; ROS at low concentrations are involved as secondary messengers in several plant hormone responses, including seed germination, lignin biosynthesis, programmed cell death, and osmotic stress, while at high concentrations ROS are known to cause oxidative damage to proteins, lipids, and DNA [81]. It has been suggested that proper regulation of ROS homeostasis is necessary for cotton fiber elongation [28], [58]. For example, many ROS genes were up-regulated in parallel in domesticated diploid and polyploid cottons during early fiber elongation (2 dpa) [82], but only a few genes were investigated during fiber elongation, including ascorbate peroxidase (*APX*) [83], copper/zinc superoxide dismutase (*CSD*) [17], and peroxidase (*POX*) [84], which were all up-regulated in domesticated cottons relative to wild cottons at 10 dpa (Table S4). H_2_O_2_ accumulated at low levels during early elongation and peaks at 20 dpa in domesticated cottons [83], [85], but its levels have not been examined in wild accessions. Interestingly, recent analysis of transcriptomic profiles of a lintless mutant compared to its wild type *G. hirsutum* at 8 and 12 dpa showed higher expression levels of genes related to stress-response processes [12]. Up-regulation of stress-related genes in the lintless mutant and wild accessions of *G. hirsutum* relative to domesticated cottons suggests elevated levels of ROS in mutant and wild cotton fibers. It would be interesting to carefully evaluate the levels of different ROS molecules during development in wild vs. cultivated cotton under controlled conditions.

### Concluding remarks

Although the transcriptomic data presented here are extraordinarily complex, as is usually the case in comparative profiling experiments, the data allow a speculative scenario to emerge from a consideration of the different classes of genes and pathways that are enriched under domestication. Specifically, we raise the suggestion that initial domestication of *G. hirsutum*, followed by several millennia of improvement and breeding, resulted in a shift or reallocation of resources from stress-related pathways in wild cottons to greater growth in domesticated forms. We envision that the reallocation and accompanying divergence in multiple pathways led to a prolonged period of fiber elongation, which at maturity are recognized now as the long, white, and fine fibers of modern cotton commerce. This scenario should become testable using a combination of forward genetic tools combined with advanced segregating populations (e.g., isogenic introgression lines), in conjunction with genomic, transcriptomic, proteomic, and metabolomic profiling. This systems approach holds the promise of improving our understanding of the evolutionary modification of a remarkable single-celled structure, while simultaneously providing clues to advance cotton breeding objectives.

## Materials and Methods

### Plant materials and library construction

Four wild and five domesticated *G. hirsutum* were selected for fiber transcriptome profiling based on their geographic origins and cotton fiber traits (Table 1; Figure 1). Wild cottons were originally from Yucatan, Mexico [86], while domesticated cottons were from four major cotton cultivation areas, i.e., Plains, Delta, and eastern and western U.S.A. Between three and twenty ovaries were collected for domesticated and wild cottons, respectively, from two developmental stages, 10 and 20 dpa, and were immediately dissected to harvest ovules, which were snap-frozen in liquid nitrogen until extraction. RNA was extracted using either a hot borate/lithium chloride procedure [87] or a CTAB extraction protocol [88], then purified by the RNeasy Plant Mini Kit (Qiagen, Stanford, CA, USA). Purified RNAs were quantified and qualified with Agilent 2100 Bioanalyzer (Agilent, Santa Clara, CA, USA). After mRNA purification using the MicroPoly(A) Purist kit (Ambion, Austin, TX, USA), RNA-Seq libraries were constructed with NEBNext mRNA Sample Prep Master Mix Set 1 following the manufacturer's suggestion (New England Biolabs, MA, USA). The constructed libraries, indexed with six nucleotide sequences, were pooled together with equimolar amounts and were sequenced on the Illumina HiSeq 2000 sequencer with 100 base reads at the Genomics Core Facility at the University of Oregon. Short read sequences were deposited in the NCBI Sequence Read Archive (SRA) with a study number SRP017061.

### Analysis of RNA-seq data: Mapping and differential expression

Raw reads were sorted into the correct accession according to their indexed nucleotides. After trimming the indexed sequences, reads were filtered based on the quality scores (Q = 20) and read length (≧17 bp) with a fastx tool kit (http://hannonlab.cshl.edu/fastx_toolkit/index.html). Fastq formatted reads were mapped to the reference genome (Cotton D V2.0; 37,505 genes) [16] using GSNAP [89]. Reads with SNP information between A and D genome progenitors were parsed into A or D homoeolog-specific bins (A_t_ or D_t_) using PolyCat (http://bioinfo3.pgml.uga.edu/polyCat/upload.html) [90].

Before identifying differentially expressed genes in each comparison of domestication (wild vs. domesticated *G. hirsutum*) and development (10 vs. 20 dpa), we examined the sample relations based on a multidimensional scale (or principal coordinate) using the edgeR package (ver. 2.0.5) in R software (ver. 2.16.0) [91]. If one sample shows a large distance from the others, that sample was removed for computing differential expression. The DESeq package (ver. 2.1.0) was used to detect differentially expressed genes in each contrast of domestication and development [92], and differential expression was defined when a gene showed at least 1.5-fold change with RPKM≧1 (RPKM: Reads Per Kilobase of gene model per Million mapped reads) [19] in all biological replicates of either wild or domesticated *G. hirsutum*. Also, to evaluate whether specific chromosomes or chromosome regions were selected during domestication and development, we investigated the distribution of differentially expressed genes on the 13 chromosomes in the haploid diploid cotton genome. For homoeolog-specific read counts, expression bias was evaluated using Fisher's exact test of the edgeR package. The distribution of *p*-values was controlled for a false discovery rate (FDR) by the BH method [93] at α = 0.05. Homoeolog-specific reads were analyzed as described in Yoo et al. [55], and differential expression was delimited by 1.5-fold expression changes with RPKM≧1 in either A_t_ or D_t_ reads across all biological replicates. In addition, we traced homoeolog expression changes during development and by domestication via comparing homoeolog expression patterns in each contrast. For example, A_t_ reads at 10 dpa were more down-regulated or highly expressed in domesticated cottons than in wild cottons, this expression change was tabulated as reflecting down- or up-regulation of A_t_, respectively, during domestication.

For several gene families where some members are known to be involved in fiber development, we examined expression patterns of individual paralogs and (homoeologs) based on their phylogenetic relationships. Sequences were annotated by homology search against *Arabidopsis thaliana* and aligned via Clustal W [94]. Phylogenetic trees were constructed using MEGA 5.05 with a default option of Maximum Parsimony [95], and majority rule consensus trees were constructed.

### Functional analysis of differentially expressed genes

To explore the nature of the biological pathways that were altered by domestication or that change during development, differentially expressed genes in each contrast were analyzed by SEA tool of agriGO (http://bioinfo.cau.edu.cn/agriGO/index.php) which performs GO term enrichment in one set of genes by comparing it to a reference list using fold changes [96]. For SEA, we used genes identified as differentially expressed (RPKM≧5 in either wild or domesticated cottons) in each contrast, with multi-test adjustment of the Benjamini-Yekutieli method (FDR<0.05) [97], and a minimum 5 mapping entries.

## Supporting Information

Figure S1 Multidimensional scaling (MDS) plot showing relationships among samples in two dimensions. Because Maxxa at 10 dpa exhibited a large distance from other domesticated cottons and was embedded in wild cottons from 10 dpa, it was removed from further analysis. Red and blue circles indicate domesticated cottons from 10 and 20 dpa, respectively, while red and blue triangles represent wild cottons from 10 and 20 dpa, respectively.
(PPTX)

Figure S2 (A) *CesA* and *Csl* gene expression patterns during development. Inside and outside circles show the expression variation in domesticated and wild cottons, respectively. Blue and red circles indicate up-regulation at 10 and 20 dpa, respectively. Grey circles denote lack of differential expression, while white circles indicate lack of expression (zero read count). (B) Expression levels of *CesA* genes. (C) Expression levels of *Csl* genes. Bars in the charts show standard errors.
(PPTX)

Figure S3 Expression patterns of *XTH* genes in developing cotton fibers. Bar in the chart shows standard error.
(PPTX)

Figure S4 A putative nuclear-mitochondrial DNA sequence block (NUMT) (red-circled area) showing the fold changes across development of domesticated cottons (red cross: log2 dom20/dom10) or between wild and domesticated cottons at 20 dpa (blue diamond: log2 dom20/wild20) on chromosome 1.
(PPTX)

Figure S5 3-ketoacyl-CoA synthase (*KCS*) gene expression patterns. (A) Phylogenetic relationships of 27 KCS genes in *G. raimondii*. Genes in red and bold were up-regulated at domesticated cottons at 10 and 20 dpa, respectively. Asterisk (*) indicates A homoeolog expression bias in domesticated cottons. (B) Gene expression levels of differentially expressed *KCS*s. The expression level of Gorai.002G218500 is shown as half of its original value to allow comparison with other genes. Bar denotes standard error.
(PPTX)

Figure S6 Phylogenetic relationship of *FLA* homologues from *Arabidopsis thaliana* and *Gossypium raimondii*. Several *FLA* homologues from *G. hirsutum* were included (e.g., *GhFLA*; Huang *et al.* 2013) and group information is based on MacMillan *et al.* (2010). Blue and red arrows indicate genes up-regulated in wild and domesticated cottons at 10 dpa, respectively.
(PPTX)

Figure S7 The expression patterns of *PROFILIN* genes and several of its partners in wild and domesticated cottons.
(PPTX)

Figure S8 Expression patterns of MYB transcription factor related to phenylpropanoid biosynthesis.
(PPTX)

Table S1 Number of differentially expressed genes in developing cotton fibers within wild and domesticated cottons using subsets of RNA-Seq data and two different techniques.
(XLSX)

Table S2 Number of differentially expressed genes in developing cotton fibers between wild and domesticated cottons using subsets of RNA-Seq data and two different techniques. Grey shaded data are from the entire data set analysis, and red text indicates the use of biological replicates from the same accession. As for RNA-Seq data, RPKM≧1 was considered for differential expression if not specified.
(XLSX)

Table S3 Chromosomal distribution of differentially expressed genes on Cotton D genome. The first table is from comparison of development, and the second one is from domestication comparison within wild and domesticated cotton. Numbers in red indicates statistically over-represented chromosome based on Chi-square test (P<0.05).
(XLSX)

Table S4 Genes related to fiber development and their expression patterns in wild and domesticated cottons. Red and bold text indicates up-regulated in wild or domesticated cottons relative to their counterparts (RPKM≧5, P<0.05). dpa = days post anthesis. RPKM = Reads Per Kilobase of gene model per Million mapped reads.
(DOCX)

Table S5 Homoeolog-specific regulation of 3-ketoacyl-CoA synthase (KCS) genes in developing cotton fiber. Expression levels were normalized with RPKM.
(XLSX)

Table S6 Transcription factors that are the most highly expressed (RPKM≧100) in developing fibers and/or which are differentially expressed between wild and domesticated cottons (bold text, P<0.05). Underlined indicates differential expression when one domesticated accession was excluded.
(XLSX)

Table S7 Comparison of results from Single Enrichment Analysis (SEA) of differentially expressed genes across two developmental stages and between wild and domesticated cottons. In ontology, P, M, and C indicate Biological Process, Molecular Function, and Cellular Components, respectively. The yellow-to-red colored blocks in CM (colorful mode) represent the level of up-regulation of each term; gray blocks indicate that the term is not significant. Numbers under CM correspond to the each comparison set, e.g., (1) shows SEA results for up-regulated at 10 dpa compared to 20 dpa in domesticated cottons.
(XLSX)
