# Supplementary material for: Comparative Evolutionary and Developmental Dynamics of the Cotton (Gossypium hirsutum) Fiber Transcriptome
Source: PLoS Genet. 2014 Jan 2;10(1):e1004073. doi: 10.1371/journal.pgen.1004073 (PMC3879233; doi:10.1371/journal.pgen.1004073)
Supplement: Table S4 — Genes related to fiber development and their expression patterns in wild and domesticated cottons. Red and bold text indicates up-regulated in wild or domesticated cottons relative to their counterparts (RPKM≧5, P<0.05). dpa = days post anthesis. RPKM = Reads Per Kilobase of gene model per Million mapped reads. (DOCX) [file pgen.1004073.s012.docx]

Table S4. Genes related to fiber development and their expression patterns in wild and domesticated cottons. Red and bold text indicates up-regulated in wild or domesticated cottons relative to their counterparts (RPKM ≧ 5, P < 0.05). dpa=days post anthesis. RPKM= Reads Per Kilobase of gene model per Million mapped reads

1. Fiber initiation

| GoraiID | Sequence description | Gene (accession no.) | Stage  (dpa) | wild.  RPKM | dom.  RPKM | Reference | Highly Expressed tissues |
| --- | --- | --- | --- | --- | --- | --- | --- |
| Gorai.012G061800 | MYB109 | GhMYB109 (AJ549758) | 10 | **32** | 23 | [[1](#_ENREF_1),[2](#_ENREF_2)] | 0~2 dpa fibers |
|  |  |  | 20 | 1 | 3 |  |  |
| Gorai.008G179600 | myb domain protein | GhMyb25-like (HM134080) | 10 | **30** | 8 | [[2](#_ENREF_2),[3](#_ENREF_3)] | -1~3 dpa fibers |
|  |  |  | 20 | **26** | 5 |  |  |
| Gorai.012G186500 | myb domain protein | GhMyb25 (EU826465) | 10 | **15** | 4 | [[2](#_ENREF_2),[4](#_ENREF_4)] | -1~2 dpa fibers |
|  |  |  | 20 | 0 | 0 |  |  |
| Gorai.004G196800 | myb domain protein | GhMYB2 (JQ868561) | 10 | **65** | 27 | [[5](#_ENREF_5)] | 0~2 dpa fibers |
|  |  |  | 20 | 23 | 23 |  |  |
| Gorai.012G061800 | myb domain protein | GhMyb109 (AJ549758) | 10 | **32** | 16 | [[1](#_ENREF_1)] | 0~3 dpa fibers |
|  |  |  | 20 | 1 | 3 |  |  |
| Gorai.009G182300 | ACC oxidase 1 (ACO1) | GhACO1 (DQ116442) | 10 | **44** | 9 | [[1](#_ENREF_1)] | 1 dpa fibers |
|  |  |  | 20 | **58** | 17 |  |  |
| Gorai.010G184900 | ACC oxidase 2 (ACO2) | GhACO2 (DQ116443) | 10 | 1 | 1 | [[1](#_ENREF_1)] | 3 dpa fibers |
|  |  |  | 20 | 1 | 1 |  |  |
| Gorai.007G150000 | indole-3-acetic acid inducible 11 | GhAUX4 (HQ452484) | 10 | 16 | 7 | [[6](#_ENREF_6)] | 0 dpa fibers |
|  |  |  | 20 | **28** | 7 |  |  |
| Gorai.004G222500 | indole-3-acetic acid inducible 11 | GhAUX5 (HQ452485) | 10 | 17 | 14 | [[6](#_ENREF_6)] | 0 dpa fibers |
|  |  |  | 20 | **24** | 13 |  |  |
| Gorai.006G246000 | indole-3-acetic acid inducible 14 | GhAUX6 (HQ452486) | 10 | 44 | 69 | [[6](#_ENREF_6)] | 0 dpa fibers |
|  |  |  | 20 | 34 | **66** |  |  |
| Gorai.013G200700.1 | indoleacetic acid-induced protein 16 | GhAUX7 (HQ452487) | 10 | 7 | 4 | [[6](#_ENREF_6)] | 0 dpa fibers |
|  |  |  | 20 | 7 | 4 |  |  |
| Gorai.010G031600 | indole-3-acetic acid inducible 9 | GhAUX8 (HQ452488) | 10 | 164 | 112 | [[6](#_ENREF_6)] | 2 dpa fibers |
|  |  |  | 20 | **290** | 95 |  |  |
| Gorai.010G177800 | protodermal factor 2 | GhHD-1 (AY464063) | 10 | **15** | 6 | [[2](#_ENREF_2)] | -1~2 dpa fibers |
|  |  |  | 20 | 2 | 1 |  |  |
| Gorai.010G037400 | Cupredoxin superfamily protein | GhBCP1 (EF222282) | 10 | 0 | 1 | [[7](#_ENREF_7)] | 3 dpa fibers |
|  |  |  | 20 | **138** | 48 |  |  |

1. Fiber elongation

| GoraiID | Sequence description | Gene (accession no.) | Stage  (dpa) | | wild.  RPKM | dom.  RPKM | Reference | Highly Expressed tissues |
| --- | --- | --- | --- | --- | --- | --- | --- | --- |
| Gorai.008G216800 | cotton vacuolar invertase | GhVIN1 (FJ915120) | 10 | | 624 | **896** | [[8](#_ENREF_8)] | 5 dpa fibers |
|  |  |  | 20 | | 71 | **289** |  |  |
| Gorai.N017200 | MADS-box transcription factor family protein | GbAGL2 (FJ198050) | 10 | | **50** | 21 | [[9](#_ENREF_9)] | 5 dpa fibers, carpels, and ovules |
|  |  |  | 20 | | **53** | 18 |  |  |
| Gorai.012G041900 | protodermal factor 1 | GbPDF1 (DQ912946) | 10 | | 3223 | 4866 | [[10](#_ENREF_10)] | 5 dpa fibers |
|  |  |  | 20 | | 293 | **1398** |  |  |
| Gorai.012G111500 | 3-oxo-5-alpha-steroid 4-dehydrogenase family protein | GhDET2 (AY141136) | 10 | | 87 | **117** | [[11](#_ENREF_11)] | 5 dpa fibers, 10 dpa ovules |
|  |  |  | 20 | | 24 | 30 |  |  |
| Gorai.009G104500 | ascorbate peroxidase 1 | GhAPX1 (EF432582) | 10 | | 597 | **869** | [[12](#_ENREF_12)] | 5 and 10 dpa fibers |
|  |  |  | 20 | | 447 | 421 |  |  |
| Gorai.012G068600 | dehydroascorbate reductase 2 | DHAR2 (AAL71857) | 10 | | **780** | 293 | [[13](#_ENREF_13)] | 5 and 10 dpa fibers |
|  |  |  | 20 | | 406 | 209 |  |  |
| Gorai.007G012000 | HXXXD-type acyl-transferase family protein | GhACY (AAL67994) | 10 | | 311 | **716** | [[13](#_ENREF_13)] | 5 and 10 dpa fibers |
|  |  |  | 20 | | 31 | **139** |  |  |
| Gorai.012G030500 | actin 7 | ACT8 (AAP73455) | 10 | | 73 | **144** | [[13](#_ENREF_13)] | 5 and 10 dpa fibers |
|  |  |  | 20 | | 79 | 86 |  |  |
| Gorai.003G131200 | beta-ketoacyl reductase 1 | GhKCR1 (AY902466) | 10 | | 75 | **154** | [[14](#_ENREF_14)] | 5 and 10 dpa fibers |
|  |  |  | 20 | | 53 | 69 |  |  |
| Gorai.003G131700 | beta-ketoacyl reductase 1 | GhKCR1 (AY902466) | 10 | | 122 | **215** | [[14](#_ENREF_14)] | 5 and 10 dpa fibers |
|  |  |  | 20 | | 63 | **77** |  |  |
| Gorai.005G173900 | beta-ketoacyl reductase 2 | GhKCR2 (AY902467) | 10 | | 65 | 70 | [[14](#_ENREF_14)] | 5 and 10 dpa fibers |
|  |  |  | 20 | | 21 | 18 |  |  |
| Gorai.005G174100 | beta-ketoacyl reductase 2 | GhKCR2 (AY902467) | 10 | | 171 | **464** | [[14](#_ENREF_14)] | 5 and 10 dpa fibers |
|  |  |  | 20 | | 20 | **153** |  |  |
| Gorai.013G192900 | copper/zinc superoxide dismutase 1 | GhCSD1 (DQ088818) | 10 | | 69 | **93** | [[15](#_ENREF_15)] | 8 dpa fibers |
|  |  |  | 20 | | 190 | 175 |  |  |
| Gorai.006G104900 | copper/zinc superoxide dismutase 2 | GhCSD2 (EU597269) | 10 | | 42 | **68** | [[15](#_ENREF_15)] | 8 dpa fibers |
|  |  |  | 20 | | 50 | 50 |  |  |
| Gorai.009G265100 | STK, AGL11 | GbAGL1 (ACI23560) | 10 | | **367** | 195 | [[16](#_ENREF_16)] | 8 dpa ovules |
|  |  |  | | 20 | **389** | 162 |  |  |
| Gorai.009G167000 | Fiber protein E6 | GhE6 (U30506) | 10 | | 9538 | 13963 | [[17](#_ENREF_17)] | 9 dpa fibers |
|  |  |  | 20 | | 6281 | 6416 |  |  |
| Gorai.007G012000 | HXXXD-type acyl-transferase family protein | GhACY (AY072824) | 10 | | 311 | **680** | [[17](#_ENREF_17)] | 9 dpa fibers |
|  |  |  | 20 | | 31 | 50 |  |  |
| Gorai.001G172900 | 2-oxoglutarate (2OG) and Fe(II)-dependent oxygenase superfamily protein | GhGA20ox1 (AY603789) | 10 | | 2 | 1 | [[18](#_ENREF_18)] | 10 dpa ovules |
|  |  |  | | 20 | 3 | 1 |  |  |

| Gorai.001G173000 | 2-oxoglutarate (2OG) and Fe(II)-dependent oxygenase superfamily protein | GhGA20ox1 (AY603789) | 10 | 3 | 1 | [[18](#_ENREF_18)] | 10 dpa ovules |
| --- | --- | --- | --- | --- | --- | --- | --- |
|  |  |  | 20 | 3 | 1 |  |  |
| Gorai.007G080700 | early nodulin-like protein 17 | GhBCP4 (GU451702) | 10 | 499 | 558 | [[7](#_ENREF_7)] | 10 dpa fibers |
|  |  |  | 20 | 102 | **216** |  |  |
| Gorai.003G088700 | Peroxidase superfamily protein | GhPOX1 (FJ415220) | 10 | 9 | **26** | [[19](#_ENREF_19)] | 10 dpa fibers |
|  |  |  | 20 | 16 | **29** |  |  |
| Gorai.012G070200 | 3-oxo-5-alpha-steroid 4-dehydrogenase family protein | GhECR1 (EU001742) | 10 | 15 | 12 | [[20](#_ENREF_20)] | 10 dpa fibers |
|  |  |  | 20 | 10 | 8 |  |  |
| Gorai.011G260500 | 3-oxo-5-alpha-steroid 4-dehydrogenase family protein | GhECR2 (EU001743) | 10 | 209 | **325** | [[20](#_ENREF_20)] | 10 dpa fibers |
|  |  |  | 20 | 86 | 111 |  |  |
| Gorai.003G157700 | NAD(P)-binding Rossmann-fold superfamily protein | GhKCR3 (AY902468) | 10 |  |  | [[21](#_ENREF_21)] | 10 dpa fibers |
|  |  |  | 20 |  |  |  |  |
| Gorai.006G119900 | FASCICLIN-like arabinogalactan-protein 12 | GhFLA1 (EF672627)  GhAGP2 (EF470296) | 10 | 1639 | **3262** | [[22](#_ENREF_22)] | 10 dpa fibers |
|  |  |  | 20 | 2262 | 3319 |  |  |
| Gorai.003G108300 | Pectin lyase-like superfamily protein | GhPEL (GQ340734) | 10 | 694 | **1168** | [[23](#_ENREF_23)] | 10 dpa fibers |
|  |  |  | 20 | 226 | 248 |  |  |
| Gorai.002G218500 | 3-ketoacyl-CoA synthase 6 | GhKCS13 (JQ922562) | 10 | 459 | **1610** | [[24](#_ENREF_24)] | 10 dpa fibers |
|  |  |  | 20 | 307 | 480 |  |  |
| Gorai.002G001100 | not KCS; GNS1/SUR4 membrane protein family | GhKCS12 | 10 | 725 | **1587** | [[24](#_ENREF_24)] | 10 dpa fibers |
|  |  |  | 20 | 288 | **509** |  |  |
| Gorai.011G212900 | annexin 8 | AnnGh4 (JX897060) | 10 | 828 | 555 | [[25](#_ENREF_25),[26](#_ENREF_26)] | 10, 12 dpa fibers |
|  |  |  | 20 | **909** | 311 |  |  |
| Gorai.007G057400 | xyloglucan endotransglucosylase/hydrolase 7 | GhXTH1 (HM749062)  GhXTH1-1 (EF546795)  GhXTH1-2 (EF546796) | 10 | 343 | **929** | [[27](#_ENREF_27),[28](#_ENREF_28)] | 15 dpa fibers |
|  |  |  | 20 | 279 | **457** |  |  |
| Gorai.011G165200 | 3-ketoacyl-CoA synthase 2 | GhKCS2 (EU001741) | 10 | 62 | **203** | [[24](#_ENREF_24)] | 15 dpa fibers |
|  |  |  | 20 | 69 | 90 |  |  |
| Gorai.007G261100 | copper/zinc superoxide dismutase 3 | GhCSD3 (EU597271) | 10 | 11 | 9 | [[15](#_ENREF_15)] | 16 dpa fibers |
|  |  |  | 20 | 15 | 7 |  |  |
| Gorai.009G102300 | 3-ketoacyl-CoA synthase 12 | GhKCS6 (EF688566) | 10 | 126 | **406** | [[24](#_ENREF_24)] | 3~10 dpa fibers |
|  |  |  | 20 | 40 | **99** |  |  |
| Gorai.009G237900 | annexin 1 | AnnGh3 (JX897059) | 10 | 1477 | 1772 | [[25](#_ENREF_25)] | 3~12 dpa fibers |
|  |  |  | 20 | 1139 | 1153 |  |  |
| Gorai.009G052200 | BURP domain-containing protein | GhRDL1 | 10 | 1319 | 1514 | [[5](#_ENREF_5)] | 3~15 dpa fibers |
|  |  |  | 20 | 624 | 810 |  |  |
| Gorai.009G052200 | BURP domain-containing protein | GhRDL (AY072821) | 10 | 1324 | 1537 | [[17](#_ENREF_17)] | 5~14 dpa fibers |
|  |  |  | 20 | 627 | 600 |  |  |
| Gorai.013G221500 | 3-ketoacyl-CoA synthase 10 | GhFDH (AY072823) | 10 | 400 | **554** | [[17](#_ENREF_17)] | 5~14 dpa fibers |
|  |  |  | 20 | 159 | 144 |  |  |
| Gorai.009G310800 | SERINE CARBOXYPEPTIDASE-LIKE 49 | GhSCP (AY072822) | 10 | 424 | **721** | [[17](#_ENREF_17)] | 5~14 dpa fibers |
|  |  |  | 20 | 323 | 320 |  |  |
| Gorai.004G210600 | beta-tubulin | GhTUB1 (AF487511) | 10 | 1140 | **2346** | [[29](#_ENREF_29)] | 8~14 dpa fibers |
|  |  |  | 20 | 1825 | 1742 |  |  |
| Gorai.003G158100 | plasma membrane intrinsic protein 1;4 | GhPIP1;3 (DQ402075) | 10 | 53 | 42 | [[30](#_ENREF_30)] | 9~12 dpa fibers |
|  |  |  | 20 | **81** | 26 |  |  |
| Gorai.007G036800 | TEOSINTE BRANCHED, cycloidea and PCF (TCP) 14 | GhTCP14 (AF165924) | 10 | 49 | **107** | [[31](#_ENREF_31)] | 6~15 dpa fibers |
|  |  |  | 20 | 8 | **11** |  |  |
| Gorai.009G016200 | indole-3-acetic acid inducible 29 | GhAUX9 (HQ452489) | 10 | **19** | 10 | [[6](#_ENREF_6)] | 10~15 dpa fibers |
|  |  |  | 20 | **43** | 11 |  |  |
| Gorai.003G103000 | AGAMOUS-like 8 | GhMADS11 (HM989877) | 10 | 2 | 2 | [[32](#_ENREF_32)] | 12~15 dpa fibers |
|  |  |  | 20 | 3 | 2 |  |  |
| Gorai.009G009500 | cellulose synthase A1 | Gh(AtCesA1-like) | 10 | 40 | **76** | [[33](#_ENREF_33)] | 9~20 dpa fibers |
|  |  |  | 20 | 47 | 47 |  |  |
| Gorai.011G005100 | Chitinase family protein | GhCHIA7 | 10 | 397 | 556 | [[33](#_ENREF_33)] | 9~20 dpa fibers |
|  |  |  | 20 | **652** | 284 |  |  |
| Gorai.008G200000 | COBRA-like extracellular glycosyl-phosphatidyl inositol-anchored protein family | Gh(AtCOB-like) | 10 | 59 | **80** | [[33](#_ENREF_33)] | 9~20 dpa fibers |
|  |  |  | 20 | 55 | 23 |  |  |
| Gorai.013G022900 | actin depolymerizing factor 4; GhADF1 | GhADF1 (DQ088156) | 10 | 150 | 130 | [[34](#_ENREF_34)] | not specified, Down-regulates organization of actin cytoskeleton; transgenic cotton |
|  |  |  | 20 | **205** | 99 |  |  |
| Gorai.001G131200 | FASCICLIN-like arabinogalactan-protein 11 | GhAGP4 (EF470295) | 10 | 1 | 2 | [[35](#_ENREF_35)] | up-regualted by GA3 in cultured ovules (1 and 2 weeks) |
|  |  |  | 20 | **683** | 6 |  |  |
| Gorai.008G155400 | FASCICLIN-like arabinoogalactan 9 | GhFLA1 (EF470298) | 10 | 546 | **1917** | [[35](#_ENREF_35)] | not affected by GA3 in cultured ovules (1 and 2 weeks) |
|  |  |  | 20 | 839 | **2157** |  |  |
| Gorai.013G256000 | FASCICLIN-like arabinogalactan-protein 12 | GhAGP3 (EF470297) | 10 | 0 | 0 | [[35](#_ENREF_35)] | up-regualted by GA3 in cultured ovules (1 and 2 weeks) |
|  |  |  | 20 | **101** | 1 |  |  |
| Gorai.012G147000 | tubulin alpha-2 chain | GhTUA9 (EF151305) | 10 | 2101 | 3004 | [[35](#_ENREF_35)] | Not affected in 9 dpa fiber of transgenic plants (GhAGP4) |
|  |  |  | 20 | 3302 | 3269 |  |  |
| Gorai.009G224800 | tubulin alpha-2 chain | GhTUA10 (EF151306) | 10 | 3707 | 3346 | [[35](#_ENREF_35)] | Not affected in 9 dpa fiber of transgenic plants (GhAGP4) |
|  |  |  | 20 | **6299** | 2621 |  |  |
| Gorai.011G128500 | expansin A8 | expansin (AY189969) | 10 | 6182 | 8443 | [[35](#_ENREF_35)] | up-regualted by GA3 in cultured ovules (1 and 2 weeks) |
|  |  |  | 20 | 4016 | 3286 |  |  |
| Gorai.007G063600 | actin 7 | GhACT2 (AY305724) GhACT5 (AY305727) | 10 | 922 | 1327 | [[36](#_ENREF_36)] | not specified |
|  |  |  | 20 | 749 | 697 |  |  |
| Gorai.003G069800 | actin 7 | GhACT4 (AY305726)  GhACT11 (AY305732) | 10 | 635 | 828 | [[36](#_ENREF_36)] | not specified |
|  |  |  | 20 | 491 | 387 |  |  |
| Gorai.013G229500 | profilin4 | GhPFN1 (AI729533) | 10 | 253 | 342 | [[37](#_ENREF_37)] | not specified |
|  |  |  | 20 | 125 | 111 |  |  |

1. Secondary wall biosynthesis

| GoraiID | Sequence description | Gene (accession no.) | Stage  (dpa) | wild.  RPKM | dom.  RPKM | Reference | Highly Expressed tissues |
| --- | --- | --- | --- | --- | --- | --- | --- |
| Gorai.011G031400 | RAC-like 2 | GhRAC13 (S79308) | 10 | 11 | 16 | [[13](#_ENREF_13),[38](#_ENREF_38)] | contribute to an oxidative burst that stimulates the onset of secondary wall deposition |
|  |  |  | 20 | **183** | 46 |  |  |
| Gorai.004G282400 | profilin3 | GhPFN2 (GU237487) | 10 | 611 | 794 | [[39](#_ENREF_39)] | At the period of transition from elongation to secondary wall deposition |
|  |  |  | 20 | 499 | 500 |  |  |
| Gorai.009G242500 | Glycosyl hydrolase superfamily protein | GhGlu1 (D88416) | 10 | 0 | 6 | [[40](#_ENREF_40)] | 20 dpa fibers |
|  |  |  | 20 | **174** | 74 |  |  |
| Gorai.009G132200 | indoleacetic acid-induced protein 16 | GhIAA16 (AJ458442) | 10 | **53** | 26 | [[6](#_ENREF_6)] | 20~23 dpa fibers |
|  |  |  | 20 | **136** | 34 |  |  |
| Gorai.006G078900 | chitinase-like protein 2 | (GhCTL1) | 10 | 1 | 1 | [[33](#_ENREF_33)] | 21~30 dpa fibers |
|  |  |  | 20 | **249** | 5 |  |  |
| Gorai.004G063600 | COBRA-like extracellular glycosyl-phosphatidyl inositol-anchored protein family | Gh(AtCOBL4-like) | 10 | 0 | 0 | [[33](#_ENREF_33)] | 21~30 dpa fibers |
|  |  |  | 20 | **130** | 3 |  |  |
| Gorai.007G374100 | FASCICLIN-like arabinogalactan-protein 11 | GhFLA6 | 10 | 1 | 2 | [[33](#_ENREF_33)] | 21~30 dpa fibers |
|  |  |  | 20 | 488 | 375 |  |  |
| Gorai.011G037900 | cellulose synthase family protein | GhCesA1 GhCESA1 (HQ143023), GhCESA4 (a copy?) | 10 | 1 | 2 | [[33](#_ENREF_33),[41](#_ENREF_41)] | 21~30 dpa fibers |
|  |  |  | 20 | **357** | 34 |  |  |
| Gorai.009G009700 | Cellulose synthase family protein | Gh(AtCesA7-like) GhCESA8 (JQ345696) | 10 | 0 | 1 | [[33](#_ENREF_33),[41](#_ENREF_41)] | 21~30 dpa fibers |
|  |  |  | 20 | **151** | 4 |  |  |
| Gorai.004G057400 | cellulose synthase A4 | Gh(AtCesA4-like) GhCESA2 (JN382210) | 10 | 3 | 4 | [[33](#_ENREF_33),[41](#_ENREF_41)] | 21~30 dpa fibers |
|  |  |  | 20 | **334** | 11 |  |  |
| Gorai.001G044700 | Cellulose synthase family protein | GhCESA7 (JQ345695) | 10 | 0 | 1 | [[41](#_ENREF_41)] | 24~29 dpa fibers |
|  |  |  | 20 | **125** | 1 |  |  |
| Gorai.013G208400 | Clathrin adaptor complexes medium subunit family protein | GhAPm (GU359054) | 10 | 11 | 8 | [[42](#_ENREF_42)] | 30 dpa fibers |
|  |  |  | 20 | 10 | 4 |  |  |
| Gorai.009G038000 | sucrose synthase 4 | GhSuSy (U73588) | 10 | 649 | 737 | [[43](#_ENREF_43)] | Not specified |
|  |  |  | 20 | 230 | 221 |  |  |
| Gorai.006G078900 | chitinase-like protein 2 | (GhCTL1) | 10 | 1 | 1 | [[33](#_ENREF_33)] | 21~30 dpa fibers |
|  |  |  | 20 | **249** | 5 |  |  |
| Gorai.004G063600 | COBRA-like extracellular glycosyl-phosphatidyl inositol-anchored protein family | Gh(AtCOBL4-like) | 10 | 0 | 0 | [[33](#_ENREF_33)] | 21~30 dpa fibers |
|  |  |  | 20 | **130** | 3 |  |  |
| Gorai.007G374100 | FASCICLIN-like arabinogalactan-protein 11 | GhFLA6 | 10 | 1 | 2 | [[33](#_ENREF_33)] | 21~30 dpa fibers |
|  |  |  | 20 | 488 | 375 |  |  |
| Gorai.011G037900 | cellulose synthase family protein | GhCesA1 GhCESA1 (HQ143023), GhCESA4 (a copy?) | 10 | 1 | 2 | [[33](#_ENREF_33),[41](#_ENREF_41)] | 21~30 dpa fibers |
|  |  |  | 20 | **357** | 34 |  |  |
| Gorai.009G009700 | Cellulose synthase family protein | Gh(AtCesA7-like) GhCESA8 (JQ345696) | 10 | 0 | 1 | [[33](#_ENREF_33),[41](#_ENREF_41)] | 21~30 dpa fibers |
|  |  |  | 20 | **151** | 4 |  |  |
| Gorai.004G057400 | cellulose synthase A4 | Gh(AtCesA4-like) GhCESA2 (JN382210) | 10 | 3 | 4 | [[33](#_ENREF_33),[41](#_ENREF_41)] | 21~30 dpa fibers |
|  |  |  | 20 | **334** | 11 |  |  |
| Gorai.001G044700 | Cellulose synthase family protein | GhCESA7 (JQ345695) | 10 | 0 | 1 | [[41](#_ENREF_41)] | 24~29 dpa fibers |
|  |  |  | 20 | **125** | 1 |  |  |
| Gorai.013G208400 | Clathrin adaptor complexes medium subunit family protein | GhAPm (GU359054) | 10 | 11 | 8 | [[42](#_ENREF_42)] | 30 dpa fibers |
|  |  |  | 20 | 10 | 4 |  |  |
| Gorai.009G038000 | sucrose synthase 4 | GhSuSy (U73588) | 10 | 649 | 737 | [[43](#_ENREF_43)] | Not specified |
|  |  |  | 20 | 230 | 221 |  |  |
|  |  |  | 10 | 0 | 6 |  | 20 dpa fibers |
|  |  |  | 20 | **174** | 74 |  |  |

References

1. Pu L, Li Q, Fan X, Yang W, Xue Y (2008) The R2R3 MYB transcription factor GhMYB109 is required for cotton fiber development. Genetics 180: 811-820.

2. Walford SA, Wu Y, Llewellyn DJ, Dennis ES (2012) Epidermal cell differentiation in cotton mediated by the homeodomain leucine zipper gene, GhHD-1. The Plant journal : for cell and molecular biology 71: 464-478.

3. Walford SA, Wu Y, Llewellyn DJ, Dennis ES (2011) GhMYB25-like: a key factor in early cotton fibre development. The Plant journal : for cell and molecular biology 65: 785-797.

4. Machado A, Wu Y, Yang Y, Llewellyn DJ, Dennis ES (2009) The MYB transcription factor GhMYB25 regulates early fibre and trichome development. The Plant journal : for cell and molecular biology 59: 52-62.

5. Guan X, Lee JJ, Pang M, Shi X, Stelly DM, et al. (2011) Activation of Arabidopsis seed hair development by cotton fiber-related genes. PloS one 6: e21301.

6. Han X, Xu X, Fang DD, Zhang T, Guo W (2012) Cloning and expression analysis of novel Aux/IAA family genes in Gossypium hirsutum. Gene 503: 83-91.

7. Ruan XM, Luo F, Li DD, Zhang J, Liu ZH, et al. (2011) Cotton BCP genes encoding putative blue copper-binding proteins are functionally expressed in fiber development and involved in response to high-salinity and heavy metal stresses. Physiologia plantarum 141: 71-83.

8. Wang L, Li XR, Lian H, Ni DA, He YK, et al. (2010) Evidence that high activity of vacuolar invertase is required for cotton fiber and Arabidopsis root elongation through osmotic dependent and independent pathways, respectively. Plant physiology 154: 744-756.

9. Liu X, Zuo K, Zhang F, Li Y, Xu J, et al. (2009) Identification and expression profile of GbAGL2, a C-class gene from Gossypium barbadense. Journal of biosciences 34: 941-951.

10. Deng F, Tu L, Tan J, Li Y, Nie Y, et al. (2012) GbPDF1 is involved in cotton fiber initiation via the core cis-element HDZIP2ATATHB2. Plant physiology 158: 890-904.

11. Luo M, Xiao Y, Li X, Lu X, Deng W, et al. (2007) GhDET2, a steroid 5alpha-reductase, plays an important role in cotton fiber cell initiation and elongation. The Plant journal : for cell and molecular biology 51: 419-430.

12. Li HB, Qin YM, Pang Y, Song WQ, Mei WQ, et al. (2007) A cotton ascorbate peroxidase is involved in hydrogen peroxide homeostasis during fibre cell development. The New phytologist 175: 462-471.

13. Yang YW, Bian SM, Yao Y, Liu JY (2008) Comparative proteomic analysis provides new insights into the fiber elongating process in cotton. Journal of proteome research 7: 4623-4637.

14. Qin YM, Pujol FM, Shi YH, Feng JX, Liu YM, et al. (2005) Cloning and functional characterization of two cDNAs encoding NADPH-dependent 3-ketoacyl-CoA reductased from developing cotton fibers. Cell research 15: 465-473.

15. Kim HJ, Triplett B (2008) Involvement of extracellular Cu/Zn superoxide dismutase in cotton fiber primary and secondary cell wall biosynthesis. Plant signaling & behavior 3: 1119-1121.

16. Liu X, Zuo KJ, Xu JT, Li Y, Zhang F, et al. (2010) Functional analysis of GbAGL1, a D-lineage gene from cotton (Gossypium barbadense). Journal of experimental botany 61: 1193-1203.

17. Li CH, Zhu YQ, Meng YL, Wang JW, Xu KX, et al. (2002) Isolation of genes preferentially expressed in cotton ﬁbers by cDNA ﬁlter arrays and RT-PCR. Plant Science 163: 1113-1120.

18. Xiao YH, Li DM, Yin MH, Li XB, Zhang M, et al. (2010) Gibberellin 20-oxidase promotes initiation and elongation of cotton fibers by regulating gibberellin synthesis. Journal of plant physiology 167: 829-837.

19. Mei W, Qin Y, Song W, Li J, Zhu Y (2009) Cotton GhPOX1 encoding plant class III peroxidase may be responsible for the high level of reactive oxygen species production that is related to cotton fiber elongation. Journal of genetics and genomics = Yi chuan xue bao 36: 141-150.

20. Song WQ, Qin YM, Saito M, Shirai T, Pujol FM, et al. (2009) Characterization of two cotton cDNAs encoding trans-2-enoyl-CoA reductase reveals a putative novel NADPH-binding motif. Journal of experimental botany 60: 1839-1848.

21. Pang Y, Song WQ, Chen FY, Qin YM (2010) A new cotton SDR family gene encodes a polypeptide possessing aldehyde reductase and 3-ketoacyl-CoA reductase activities. Biochemistry Biokhimiia 75: 320-326.

22. Huang GQ, Gong SY, Xu WL, Li W, Li P, et al. (2013) A fasciclin-like arabinogalactan protein, GhFLA1, is involved in fiber initiation and elongation of cotton. Plant physiology 161: 1278-1290.

23. Wang H, Guo Y, Lv F, Zhu H, Wu S, et al. (2010) The essential role of GhPEL gene, encoding a pectate lyase, in cell wall loosening by depolymerization of the de-esterified pectin during fiber elongation in cotton. Plant molecular biology 72: 397-406.

24. Qin YM, Hu CY, Pang Y, Kastaniotis AJ, Hiltunen JK, et al. (2007) Saturated very-long-chain fatty acids promote cotton fiber and Arabidopsis cell elongation by activating ethylene biosynthesis. The Plant cell 19: 3692-3704.

25. Li B, Li DD, Zhang J, Xia H, Wang XL, et al. (2013) Cotton AnnGh3 Encoding an Annexin Protein is Preferentially Expressed in Fibers and Promotes Initiation and Elongation of Leaf Trichomes in Transgenic Arabidopsis. Journal of integrative plant biology.

26. Huang Y, Wang J, Zhang L, Zuo K (2013) A cotton annexin protein AnxGb6 regulates fiber elongation through its interaction with actin 1. PloS one 8: e66160.

27. Lee J, Burns TH, Light G, Sun Y, Fokar M, et al. (2010) Xyloglucan endotransglycosylase/hydrolase genes in cotton and their role in fiber elongation. Planta 232: 1191-1205.

28. Michailidis G, Argiriou A, Darzentas N, Tsaftaris A (2009) Analysis of xyloglucan endotransglycosylase/hydrolase (XTH) genes from allotetraploid (Gossypium hirsutum) cotton and its diploid progenitors expressed during fiber elongation. Journal of plant physiology 166: 403-416.

29. Li XB, Cai L, Cheng NH, Liu JW (2002) Molecular characterization of the cotton GhTUB1 gene that is preferentially expressed in fiber. Plant physiology 130: 666-674.

30. Li DD, Ruan XM, Zhang J, Wu YJ, Wang XL, et al. (2013) Cotton plasma membrane intrinsic protein 2s (PIP2s) selectively interact to regulate their water channel activities and are required for fibre development. The New phytologist 199: 695-707.

31. Wang MY, Zhao PM, Cheng HQ, Han LB, Wu XM, et al. (2013) The Cotton Transcription Factor TCP14 Functions in Auxin-Mediated Epidermal Cell Differentiation and Elongation. Plant physiology 162: 1669-1680.

32. Li Y, Ning H, Zhang Z, Wu Y, Jiang J, et al. (2011) A cotton gene encoding novel MADS-box protein is preferentially expressed in fibers and functions in cell elongation. Acta biochimica et biophysica Sinica 43: 607-617.

33. Betancur L, Singh B, Rapp RA, Wendel JF, Marks MD, et al. (2010) Phylogenetically distinct cellulose synthase genes support secondary wall thickening in arabidopsis shoot trichomes and cotton fiber. Journal of integrative plant biology 52: 205-220.

34. Wang HY, Wang J, Gao P, Jiao GL, Zhao PM, et al. (2009) Down-regulation of GhADF1 gene expression affects cotton fibre properties. Plant biotechnology journal 7: 13-23.

35. Li Y, Liu D, Tu L, Zhang X, Wang L, et al. (2010) Suppression of GhAGP4 gene expression repressed the initiation and elongation of cotton fiber. Plant cell reports 29: 193-202.

36. Li XB, Fan XP, Wang XL, Cai L, Yang WC (2005) The cotton ACTIN1 gene is functionally expressed in fibers and participates in fiber elongation. The Plant cell 17: 859-875.

37. Wang HY, Yu Y, Chen ZL, Xia GX (2005) Functional characterization of Gossypium hirsutum profilin 1 gene (GhPFN1) in tobacco suspension cells. Characterization of in vivo functions of a cotton profilin gene. Planta 222: 594-603.

38. Potikha TS, Collins CC, Johnson DI, Delmer DP, Levine A (1999) The involvement of hydrogen peroxide in the differentiation of secondary walls in cotton fibers. Plant physiology 119: 849-858.

39. Wang J, Wang HY, Zhao PM, Han LB, Jiao GL, et al. (2010) Overexpression of a profilin (GhPFN2) promotes the progression of developmental phases in cotton fibers. Plant & cell physiology 51: 1276-1290.

40. Ruan YL, Xu SM, White R, Furbank RT (2004) Genotypic and developmental evidence for the role of plasmodesmatal regulation in cotton fiber elongation mediated by callose turnover. Plant physiology 136: 4104-4113.

41. Li A, Xia T, Xu W, Chen T, Li X, et al. (2013) An integrative analysis of four CESA isoforms specific for fiber cellulose production between Gossypium hirsutum and Gossypium barbadense. Planta 237: 1585-1597.

42. Zhou T, Zhang R, Yang D, Guo S (2011) Molecular cloning and characterization of GhAPm, a gene encoding the mu subunit of the clathrin-associated adaptor protein complex that is associated with cotton (Gossypium hirsutum) fiber development. Molecular biology reports 38: 3309-3317.

43. Ruan YL, Llewellyn DJ, Furbank RT (2003) Suppression of sucrose synthase gene expression represses cotton fiber cell initiation, elongation, and seed development. The Plant cell 15: 952-964.
